# Comparative analysis of supervised and self-supervised learning with small and imbalanced medical imaging datasets

**DOI:** 10.1038/s41598-025-99000-0

**Published:** 2025-09-02

**Authors:** Andrea Espis, Chiara Marzi, Stefano Diciotti

**Affiliations:** 1 Department of Electrical, Electronic, and Information Engineering “Guglielmo Marconi” – DEI, University of Bologna, Via dell’Università 50, 47521 Cesena, Italy; 2 Department of Statistics, Computer Science and Applications “Giuseppe Parenti”, University of Florence, 50134 Florence, Italy; 3 Alma Mater Research Institute for Human-Centered Artificial Intelligence, University of Bologna, 40121 Bologna, Italy

**Keywords:** Comparative study, Contrastive learning, Medical imaging, Self-supervised learning, Small datasets, Supervised learning, Biomedical engineering, Computer science

## Abstract

Self-supervised learning (SSL) in computer vision has shown its potential to reduce reliance on labeled data. However, most studies focused on balanced, large, broad-domain datasets like ImageNet, whereas, in real-world medical applications, dataset size is typically limited. This study compares the performance of SSL versus supervised learning (SL) on small, imbalanced medical imaging datasets. We experimented with four binary classification tasks: age prediction and diagnosis of Alzheimer’s disease from brain magnetic resonance imaging scans, pneumonia from chest radiograms, and retinal diseases associated with choroidal neovascularization from optical coherence tomography with a mean size of training sets of 843 images, 771 images, 1,214 images, and 33,484 images, respectively. We tested various combinations of label availability and class frequency distribution, repeating the training with different random seeds to assess result uncertainty. In most experiments involving small training sets, SL outperformed the selected SSL paradigms, even when a limited portion of labeled data was available. Our findings highlight the importance of carefully selecting learning paradigms based on specific application requirements, which are influenced by factors such as training set size, label availability, and class frequency distribution.

## Introduction

Artificial intelligence (AI) has shown significant promise in advancing healthcare^1^. Deep learning, a subset of machine learning, excels in tasks such as medical image analysis, diagnostic prediction, and treatment planning. Among deep learning models, convolutional neural networks (CNNs) are especially effective, achieving high accuracy in classification, detection, and segmentation tasks^2^. These models are predominantly trained using supervised learning (SL), which relies on labeled datasets^3^. A key advantage of deep learning over traditional machine learning approaches is its capacity to automatically learn task-relevant features from data^3^. This capability often leads to superior performance to traditional machine learning, especially in the analysis of imaging and textual data^4^. However, deep models trained with SL necessitate large volumes of labeled training data^5^, and the labeling process is generally costly and prone to errors^6^. This challenge is particularly pronounced in medical contexts, where expert data labeling is often highly time-consuming because each label may result from extensive data analysis^7^.

To mitigate these constraints, self-supervised learning (SSL)^8^ has emerged as a promising approach that reduces dependence on labeled data by leveraging the inherent structure and patterns within unlabeled data. This technique has recently gained significant attention in computer vision due to its ability to reduce the burden of manual annotation and enable more scalable and cost-effective AI solutions^9^. Although most research in this field focuses on evaluating the performance of the domain-agnostic SSL paradigm on large, natural, and balanced benchmark datasets such as ImageNet^10^ and CIFAR-10^11^, its applicability in more challenging scenarios remains underexplored. In particular, SSL has been shown to suffer performance degradation when dealing with imbalanced datasets^12,13^. This raises important questions about whether such a decline is more pronounced than for SL, especially when large amounts of data are unavailable. Furthermore, it is still unclear whether SSL outperforms SL when applied to partially labeled, small datasets—particularly when the pre-training phase relies on the same dataset as the downstream task rather than leveraging large external datasets.

To better understand these limitations, in this study, we conducted a comparative analysis of randomly initialized models trained with both learning strategies on four binary medical imaging classification tasks: age prediction and diagnosis of Alzheimer’s disease from brain magnetic resonance imaging (MRI) scans, pneumonia from chest radiograms, and retinal diseases associated with choroidal neovascularization (CNV) from optical coherence tomography (OCT). For each dataset, we experimented with different learning strategies, described in Section “Materials and methods”, and combinations of label availability and class-frequency distribution. We repeated the pre-training and fine-tuning with different random seeds to estimate the uncertainty for all datasets. Details about the preprocessing and data augmentation, the model’s architecture, and the optimization techniques used in the experiments are described respectively in Sections “Preprocessing and data augmentation”, “Models’ architectures”, and “Models’ optimization”. As described in Section “Validation scheme”, when assuming 100% label availability, the average training set size across experiments was 843 images for age prediction, 771 images for Alzheimer’s diagnosis, 1,214 images for pneumonia diagnosis, and 33,484 images for diagnosis of retinal diseases associated with choroidal neovascularization. This relatively larger dataset was included to examine the influence of training set size on the performance of different learning paradigms. Hyperparameters and configuration settings are detailed in Section “Hyperparameters and configuration settings”, while Section “Validation scheme” describes the analysis of the results, which are presented in Section “Results”. Finally, Sections “Discussion”, “Limitations”, and “Conclusion” provide, respectively, a discussion of the results, the study’s limitations, and the conclusions.

Our contributions to the field of 2D medical imaging analysis tasks are multifaceted. First, we systematically compare SL and SSL in medical image classification, addressing key challenges such as class imbalance, limited data, and computational constraints—common issues in real-world medical settings. Additionally, we emphasize the importance of avoiding methodological bias by ensuring a fair comparison by equipping both SL and SSL approaches with identical data augmentations and training procedures. We also promote reproducibility through extensive repeated experiments complemented by rigorous statistical analyses. Finally, our study provides valuable insights for AI practitioners in the medical field, allowing them to determine when SSL may be a preferable alternative to SL based on the training set size, label availability, and class frequency distribution.

## Related work

### Supervised and self-supervised learning

SSL is an unsupervised learning approach that extracts meaningful information directly from data without relying on ground-truth labels, unlike weakly supervised learning, which typically incorporates partial labeling or auxiliary supervision^14^. By leveraging patterns and structures inherent in the data, SSL learns representations that can be effectively transferred to downstream tasks. In medical imaging, SSL has demonstrated superior performance over traditional SL when performing large-scale pre-training^7^. This advantage stems from its ability to capture rich feature representations without needing extensive labeled datasets, which are often costly and time-consuming to obtain in the medical domain. Among the various applications of SSL in medical imaging, classification and segmentation are the most actively explored^15^. While SSL has been successfully applied to classification tasks, which is the focus of our study, its impact on segmentation is also noteworthy. State-of-the-art segmentation methods often integrate SSL with other learning paradigms, most commonly semi-supervised learning, to enhance performance, especially in scenarios with limited labeled data. This combination enables models to use both labeled and unlabeled images better, improving generalization and robustness in real-world medical applications^16–24^.

Prior studies have also explored the performance of SSL in medical imaging classification. Wolf et al.^25^ compared three contrastive methods, i.e., MoCo^26^, SwAV^27^, and BYOL^28^, and a masked autoencoder, SparK^29^. They pre-trained the models on 244,527 unlabeled CT slices extracted from lung CT scans of 1,010 patients, then fine-tuned with SL on different relatively small datasets from related domains: brain hemorrhages dataset they collected, the COVID-19 CT Classification Grand Challenge^30^, and the OrganSMNIST Challenge from MedMNIST^31^, with a training set of respectively 145, 425 and 13,952 images. They showed that all pre-training methods yielded significantly better results than training the model with a random initialization without pre-training. Azizi et al.^32^ investigated the benefits of pre-training using natural images (ImageNet^10^) and unlabeled medical data. They evaluated this approach with a dermatology dataset^33^ for skin condition classification (12,306 patients with one to six images per case) and a chest X-ray classification task^34^ with 14 possible outcomes (consisting of 224,316 radiographs from 65,240 patients). Both datasets exhibited significant class imbalance. Further studies extended this investigation^7^ to four additional medical tasks: diabetic macular edema classification (2,287,716 fundus images from 308,507 patients), colorectal-cancer-survival prediction (4,496 stage II and III colorectal cancer cases, 36,841 slides), mammography classification (89,018 cases), and metastases detection (10,705 cases and 29,018 slides). While these studies indicate that SSL outperforms SL despite imbalanced pre-training datasets, they do not systematically explore varying class frequency distributions. Assran et al. demonstrated that state-of-the-art SSL paradigms, including VICReg^35^, SwAV^27^, MSN^36^, and SimCLR^37^, inherently learn features that facilitate uniform clustering of data^13^. As a result, these paradigms are well-suited for balanced datasets such as ImageNet. However, their performance diminishes when pre-training on class-imbalanced data, highlighting a significant limitation in their application to diverse medical datasets.

However, despite being compromised by class imbalance, we question whether SSL performance still surpasses that of SL. In this context, Liu et al. revealed that MoCo v2^38^ and SimSiam^39^ are more robust to class imbalance than SL representations—the performance gap between balanced and imbalanced pre-training with SSL was notably smaller compared to SL^40^. They evaluated class imbalance ratios of {1, 0.004, 0.0025} for ImageNet and {1, 0.1, 0.01} for CIFAR-10, and downsampling ratios of {0.75, 0.5, 0.25, 0.125} to create datasets of varying sizes. The robustness of SL and SSL to class imbalance was compared by calculating the performance gap $\Delta^{SL}$, and $\Delta^{SSL}$, respectively, between models pre-trained on balanced versus imbalanced datasets of the same sizes under various configurations. They concluded that, under the conditions of their experiments, SSL is more robust to dataset imbalance:

1 $$\Delta^{SSL} \left( {N,r} \right) \triangleq \frac{{A^{SSL} \left( {n,1} \right) - A^{SSL} \left( {n,r} \right) }}{{A^{SSL} \left( {n,1} \right)}} \ll \Delta^{SL} \left( {N,r} \right) \triangleq \frac{{A^{SL} \left( {n,1} \right) - A^{SL} \left( {n,r} \right) }}{{A^{SL} \left( {n,1} \right)}}$$

where *N* is the pre-training sample size, A is the accuracy, and r is the ratio between the probability of the rarest class and the most frequent class:

2 $$r = \frac{{\min_{j \in \left[ C \right]} P\left( {y = j} \right)}}{{\max_{j \in \left[ C \right]} P\left( {y = j} \right)}} \le 1$$

and $P$ is a pre-training distribution over ${\mathbb{R}}^{d} \times \left[ C \right]$, where ${\mathbb{R}}^{d}$ is the space of the input samples, and C is the set of classes.

To the best of our knowledge, the only study investigating the robustness of SSL to class imbalance in the medical field was conducted by Zhang et al.^41^. They examined robustness in a classification task for the lung nodule false positive reduction in the LUNA 2016 dataset^42^, where the positive class indicated a nodule’s presence. The paradigms used for pre-training were Model Genesis^43^ and PCRL^44^, compared with models “from-scratch” without pre-training. They experimented with imbalance ratios of {0.025, 0.05, 0.075, 0.1, 0.15} and training sample size *N* of {5,000; 10,000; 150,000}. They observed that “SSL pre-training performs poorly (even worse than baseline) on extremely imbalanced and relatively balanced cases”. Nevertheless, they noted that SSL boosts the performance of rare classes. Their study focused on comparing SSL and SL but did not compare the paradigms with the performance on balanced cases ($r$ = 1), and the training set was relatively large (*N* ≥ 5,000).

While our study shares certain similarities with the works cited in this section, it is complementary due to several key differences—some of which may appear individually in prior studies, but not all together. Whereas previous work has demonstrated the effectiveness of SSL using large unlabeled datasets for pre-training different from the one used for fine-tuning^7,25,32,40,41^, our study specifically investigates whether SSL can still offer benefits in more constrained scenarios, such as using the same dataset for both pre-training and fine-tuning. This approach has two possible implications: it could show that SSL remains effective even when limited to a single dataset, or it could further justify the time and effort required to leverage larger unlabeled datasets from other domains. In contrast to studies that compare only different SSL methods^25^, our primary focus was the comparison between SSL and SL. Additionally, although some works involve imbalanced datasets^7,25,32^, they did not explicitly design experiments to systematically assess the impact of class imbalance, or they did not focus on medical imaging^40^.

### Self-contrastive learning

In our study, we focused on self-contrastive learning because it has been empirically shown to be usually more performant than other SSL paradigms when dealing with classification tasks in the medical field^45,46^. Self-contrastive learning leverages data augmentation to create variants of a single input sample using transformations that preserve semantics. These variant inputs form positive pairs, while unrelated samples are considered negative. The goal is to train a model to encode positive pair samples similarly, ignoring non-semantic differences. Paradigms like SimCLR^37^ use negative pairs to prevent the model from encoding all inputs the same way, i.e., the “model collapse”^47^. SimCLR does not need a specialized architecture like AMDIM^48^ and CPC v2^49^, or a memory bank like PIRL^50^, CMC^51^, or a dynamic dictionary like MoCo^26^. Differently, BYOL^28^ and DINO^52^ avoid collapse using self-distillation and asymmetric architectures, where the parameters of one branch are updated by the other’s exponential moving average.

However, our study focused on paradigms particularly well-suited for scenarios with constrained computational resources, ensuring their applicability in real-world medical settings. Some recent works show how the triplet loss can be used to avoid representation collapse while requiring smaller batch sizes and reduced computational power compared to SimCLR and other self-contrastive state-of-the-art paradigms^53–55^. To deal with the collapse, BarlowTwins^56^ and VICReg^35^ introduced a loss regularization term that eliminates the need for negative pairs. In particular, the VICReg loss function is defined as

3 $$l\left( {Z,Z^{\prime}} \right) = \lambda s\left( {Z,Z^{\prime}} \right) + \mu \left[ {v\left( Z \right) + v\left( {Z^{\prime}} \right)} \right] + \beta \left[ {c\left( Z \right) + c\left( {Z^{\prime}} \right)} \right]$$

where $Z = [z_{1}$,…,$z_{b} ]$, and $Z^{\prime} = [z^{\prime}_{1} , \ldots ,z^{\prime}_{b} ]$ indicate the two batches of $b$ embedding vectors, where $b$ is the batch size, i.e., the output of the two branches of the siamese architecture. The invariance term $s$ is the mean-squared Euclidean distance between each pair of vectors embedding augmented versions of the same image Z and Z′, without any normalization:

4 $$s\left( {Z,Z^{\prime}} \right) = \frac{1}{b}\mathop \sum \limits_{i} z_{i} - z_{i2}^{\prime 2}$$

The variance regularization term $v$ is a hinge function on the standard deviation of the embeddings along the batch dimension, i.e., it is the term that eliminates the need for negative pairs to prevent the collapse by forcing variability of the sample representation within the batch. Finally, the covariance regularization term $c$ de-correlates the different dimensions of the embeddings, preventing them from encoding similar information. This term is not necessary to avoid collapse; it is instead thought to improve the results of the classification downstream task by maximizing the representation of information content. In practice, the VICReg loss function is a weighted average of an invariance, variance, and covariance term weighted by the hyperparameters ${\uplambda },{ }\mu$, and $\beta$. One fundamental difference between Barlow Twins and VICReg is that VICReg applies the covariance term to each branch separately, allowing the two branches to have different types of architectures.

In our study, we selected VICReg to investigate the robustness of self contrastive class imbalance compared to SL as it is better suited for scenarios with limited GPU RAM since, as discussed above, VICReg avoids model collapse through variance regularization rather than relying on negative pairs.

### Self-prediction and generative learning

Self-prediction and generative learning are another group of state-of-the-art SSL paradigms improving models’ performance in medical imaging classification tasks^45^. Both approaches rely on reconstructing the input; the key difference is that generative learning involves the reconstruction of the whole input, while self-prediction applies masking or augmentations and requires the model to reconstruct part of the input based on the contextual information. Generative models are typically based on autoencoders and, more recently, on Generative Adversarial Networks (GANs), and there are various successful applications in the medical field^57–59^.

Self-prediction was originally proposed in the field of Natural Language Processing (NLP)^60^, with the name Masked Language Modeling (MLM) approach by predicting missing words in a sentence. Similarly, when applied to predicting the missing portion of an image, the approach is called Masked Image Modeling (MIM)^61^. For this work, we selected the Masked Autoencoder (MAE) paradigm^62^, which employs a simple mean squared error loss between the reconstructed and original images in pixel space, due to its state-of-the-art performance and its suitability for training a Vision Transformer backbone with limited GPU RAM, thanks to its efficient masking strategy. Additionally, MAE is expected to be robust to class frequency imbalance, as it does not rely on explicitly computed mini-batch statistics^13^.

## Materials and methods

This cross-sectional study utilized publicly available, de-identified datasets. Ethical approval and informed consent were obtained locally by the original investigators for each study. Additionally, data usage for ADNI complies with the guidelines and regulations established by the ADNI consortium (https://adni.loni.usc.edu/). All methods were conducted in accordance with relevant guidelines and regulations, including the Declaration of Helsinki.

### Datasets

#### HEALTHY-LIFESPAN: meta-dataset of healthy subjects

We gathered T1-weighted brain MRI scans from 1,787 healthy individuals aged 5 to 87 years across 36 single-center datasets derived from various studies, including Autism Brain Imaging Data Exchange (ABIDE) I and II^63–65^, Information eXtraction from Images (IXI)^66^, 1000 Functional Connectomes Project (FCP)^67^, and Consortium for Reliability and Reproducibility (CoRR)^68^. Each dataset utilized the same scanner and acquisition protocol at a specific location (see Table S1 for details). ABIDE I and II contributed a total of 17 datasets, labeled with prefixes such as ABIDEI or ABIDEII followed by the collecting institution (e.g., ABIDEI-CALTECH, ABIDEII-BNI_1)^69^. For consistency, datasets like LEUVEN_1 and LEUVEN_2 were merged into ABIDEI-LEUVEN, UCLA_1 and UCLA_2 into ABIDEI-UCLA, and UM_1 and UM_2 into ABIDEI-UM, based on identical acquisition parameters. Additionally, ABIDEII-KKI_1 data were divided into ABIDEII-KKI_8ch and ABIDEII-KKI_32ch due to different acquisition settings (8-channel or 32-channel phased-array head coil). The IXI study provided three datasets labeled with the prefix IXI followed by the institution’s name (e.g., IXI-Guys). From the 1000 FCP and CoRR studies, we utilized datasets such as the International Consortium for Brain Mapping (ICBM) and the Nathan Kline Institute - Rockland Sample Pediatric Multimodal Imaging Test-Retest Sample (NKI2). Each single-center dataset included baseline MRI scans of typically developing and aging brains (one per subject), along with age and sex information. Subjects were classified as having normal development and aging based on the absence of recognized neurological or psychiatric disorders. Table S1 outlines the characteristics of each single-center dataset. We aggregated these datasets into a meta-dataset named HEALTHY-LIFESPAN, covering the entire age range from 5 to 87 years.

To convert the age prediction task into a binary classification task, we used a threshold equal to 20 years (i.e., subjects with age ≤ 20 years defined the “children and adolescent” group, while subjects with age > 20 years were placed in the “adults and elders” category). The threshold has been chosen to obtain a balanced class distribution of 50%-50% and maximize the use of the data while imposing different class frequencies for the experiments with class-imbalanced configurations (see Section "Validation scheme").

#### ADNI: meta-dataset of patients with Alzheimer’s disease and cognitively normal controls

For this study, we used data from the public ADNI dataset, which can be freely accessed through the LONI Image and Data Archive (IDA) research data repository. We included the baseline and longitudinal data from all project phases (ADNI1, ADNI GO, ADNI2, ADNI3) and downloaded all T1-weighted MR images with the following characteristics: 3D acquisition type, Research Group equal to “AD” and “CN”, image descriptions included “MP-RAGE”, “MP- RAGE REPEAT”, “MP-RAGE SENSE2”, “Accelerated Sagittal MP-RAGE”, “Repeat Accelerated Sagittal MP-RAGE”, “MP-RAGE 24 FOV” and “MP-RAGE 24 FOV REPEAT”. This resulted in a total of 2,727 3D images (volumes) of 667 subjects (173 with AD and 494 CN, 298 males and 369 females, ages ranging from 51 to 96 years, mean age 76.30 years, standard deviation 6.98 years). We carefully reviewed the dataset and discarded three duplicate volumes and eight volumes corrupted by artifacts. As a result, we obtained a final dataset consisting of 2,716 volumes (789 belonging to AD patients and 1,927 to CN subjects) to train and test the model (please refer to the Supplemental files for a detailed report of correspondent Image Data (IDs)).

#### PneumoniaMNIST: meta-dataset of patients with pneumonia and healthy controls

We conducted experiments using pediatric chest radiograms from the PneumoniaMNIST dataset, a subset of the publicly available MedMNIST+ meta-dataset^70^. PneumoniaMNIST comprises 5,856 images designed for the diagnosis of pneumonia (i.e., a binary classification task: pneumonia vs. healthy controls). The dataset is pre-split into training (4,708 samples), validation (524 samples), and test sets (624 samples), ensuring no data leakage by including all images of a subject within the same split. The original radiograms had variable sizes within (384 − 2,916) × (127 − 2,713) pixels. They were center-cropped with a window size of the length of the short edge and resized to 224 × 224 pixels. The chest X-ray images were selected from retrospective cohorts of pediatric (one to five years old) patients from Guangzhou Women and Children’s Medical Center, Guangzhou. All chest X-ray imaging was performed as part of patients’ routine clinical care. For further information on the source dataset, please refer to MedMNIST v2^31^ and the original study^71^.

#### OCTMNIST: meta-dataset of patients with retinal diseases associated with choroidal neovascularization and healthy controls

We performed experiments with part of OCTMNIST—a benchmark medical imaging dataset consisting of OCT scans of the retina, categorized into multiple classes for automated diagnosis of retinal diseases associated with choroidal neovascularization. We considered only the two most frequent classes, i.e., “normal retina and retina with choroidal neurovascularization”. The selected subset is pre-split into training (79,510 samples), validation (8,835 samples), and test sets (500 samples), ensuring no data leakage by including all images of a subject within the same split. The source images had variable size, i.e., (384 − 1,536) × (277 − 512) pixels. They were center-cropped with a window size of length of the short edge and resized to 224 × 224 pixels. The images were selected from retrospective cohorts of adult patients from the Shiley Eye Institute of the University of California San Diego, the California Retinal Research Foundation, Medical Center Ophthalmology Associates, the Shanghai First People’s Hospital, and Beijing Tongren Eye Center between July 1, 2013 and March 1, 2017. All OCT imaging was performed as part of patients’ routine clinical care. For further information on the source dataset, please refer to MedMNIST v2^31^ and the original study^71^.

### Preprocessing and data augmentation

#### MR image preprocessing for model’s training

We registered each T1-weighted volume from the HEALTHY-LIFESPAN or ADNI datasets to the MNI152 standard template space with a voxel size of 1 mm^3^ using FSL^72^ (version 6.0). This registration process involved linear transformation with 9 degrees of freedom and trilinear interpolation using FSL’s FLIRT. After registration, the T1-weighted volumes had different data matrix sizes and voxel dimensions of 1 mm × 1 mm × 1 mm. To transform the field-of-view to a cubic shape with a data matrix size of 256 × 256 × 256 voxels, each registered volume was processed using Freesurfer’s *mri_convert –conform*^73^. Additionally, we adjusted the orientation of the volumes from Left-Inferior-Anterior (LIA) to Right-Anterior–Superior (RAS) to enhance visualization. Due to poor registration quality, we excluded 15 subjects from the HEALTHY-LIFESPAN meta-dataset. Next, we conducted skull stripping using FSL’s BET and extracted the central axial slice (corresponding to slice number 127) from each registered and skull-stripped T1-weighted volume. These axial slices from each meta-dataset (HEALTHY-LIFESPAN or ADNI) were then concatenated into a single NIfTI volume with dimensions of 256 × 256 × *n*, where *n* represents the total number of subjects in each meta-dataset.

#### Data augmentations

In the pre-training phase, we adopted data augmentations inspired by previous studies^74,75^ using parameter values set by visually observing their effect on the images to ensure that the semantics were preserved. Specifically, we applied cropping with output shape 224 × 224, isotropic scaling in the range (0.85, 1.15), translation in the range (− 5, 5) pixels in horizontal and vertical directions, rotation in the range (− 15, 15) degrees, random bias field artifact with maximum magnitude of polynomial coefficients sampled from the range (− 0.05, 0.05), change of contrast with the gamma correction with the ln of gamma sampled from the range (ln(0.85), ln(1.15))^75^ and additive gaussian noise with mean 0 and standard deviation 0.1.

In the fine-tuning phase, for the training set, we applied cropping with output shape 224 × 224, translation in the range (− 5, 5) pixels in horizontal and vertical directions, and rotation in the range (− 10, 10) degrees. For the test set, we applied only cropping with an output shape of 224 × 224.

### Models’ architectures

For the sake of a fair comparison, the models pre-trained with SL and VICReg were implemented using the same architecture, differing only in the final layer. They were composed by a backbone, namely randomly initialized ResNet-34^76^ truncated after the last convolutional block (layer 4), with the output processed by an adaptive average pooling layer and flattened into a 512-dimensional feature vector followed by a projector, namely two consecutive randomly initialized linear layers. The first linear layer had 512 input nodes, the intermediate 1,024, and the number of output nodes depended on the paradigm’s loss: SL loss requires just one output node for the binary classification, while VICReg output dimension is arbitrary because the loss compares the output nodes of the two backbones, thus we kept it to the same dimension of the first linear layer as done by default in VICReg original study^35^. The model pre-trained with MAE was an asymmetric autoencoder composed of 12 standard Vision Transformer (ViT) blocks^77^ for the encoder and 4 for the decoder.

The models’ architecture pre-trained with VICReg and MAE were implemented using the official code repository^78,79^. Thus, the exact same model architecture used in this study can be reproduced by setting the hyperparameters as described in Section “Hyperparameters and configuration settings”.

### Models’ optimization

#### Pre-training optimization

**Fig. 1:**
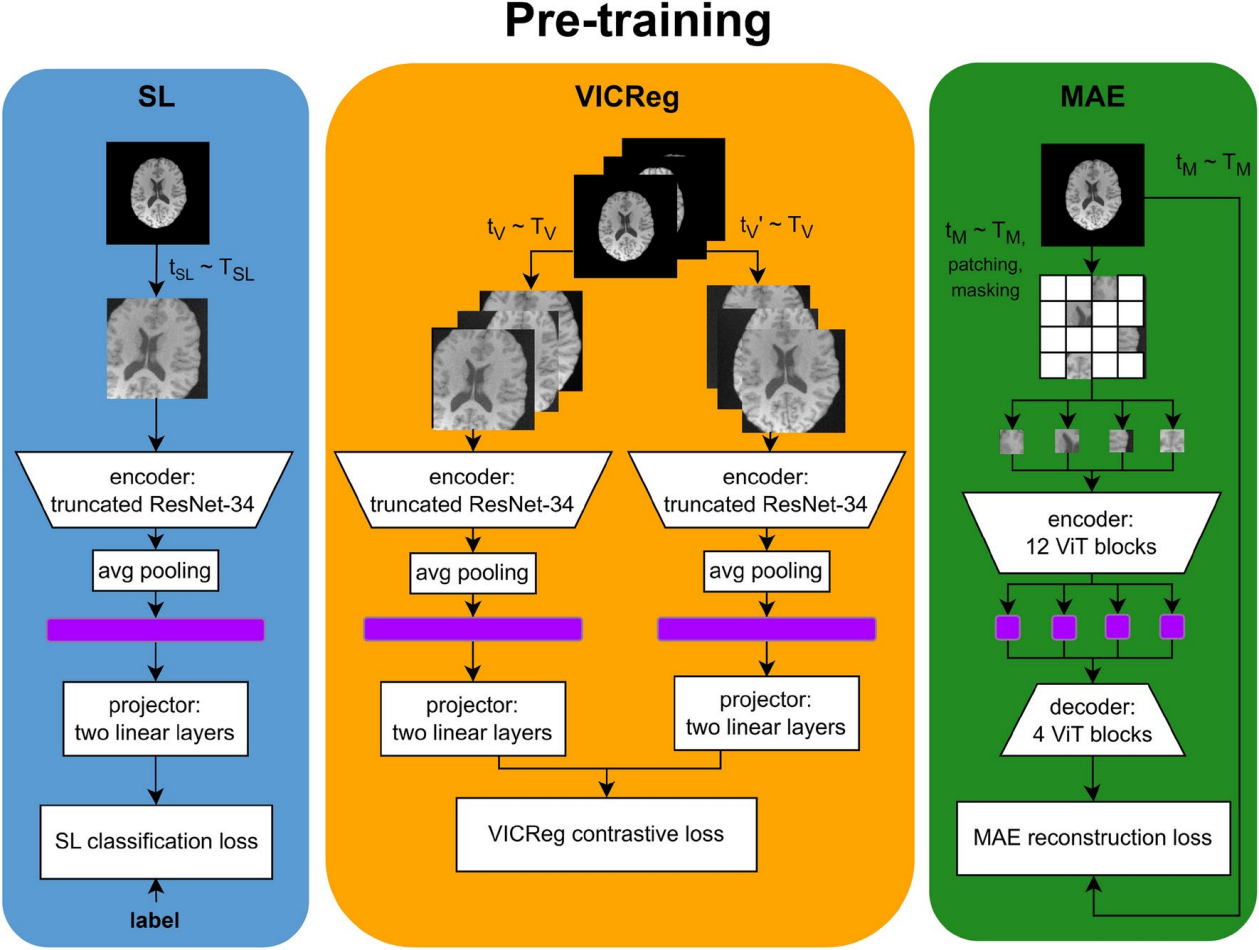
Pre-training workflow for SL, VICReg, and MAE. T is the distribution of random transformations described in Section “Data augmentations”. During the pre-training, for each batch iteration, a set of transformations ${t}_{i}$ is sampled from ${T}_{i}$, where $i$ is specific to the learning paradigm and applied to the batch of images. The figure emphasizes that only VICReg loss needs the batch to contain more than one image, and it shows brain MRI images as an example, but the same schema applies to chest radiograms or OCT images. (MAE, Masked Autoencoder; MRI, Magnetic Resonance Imaging; OCT, Optical Coherence Tomography; SL, Supervised Learning; VICReg, Variance-Invariance-Covariance Regularization).

Figure 1 schematizes the pre-training process of the models according to the SL, VICReg, and MAE paradigms. In SL, the pre-training objective is a binary classification task, distinguishing between the following class pairs for each dataset: ‘adults vs. children and adolescents’ (HEALTHY-LIFESPAN meta-dataset), ‘patients with Alzheimer’s disease vs. cognitively normal subjects’ (ADNI meta-dataset), ‘patients with pneumonia vs. healthy controls’ (PneumoniaMNIST dataset), and ‘patients with retinal diseases associated with choroidal neovascularization vs. healthy controls’ (OCTMNIST dataset). The pre-training loss function for SL was a weighted binary cross-entropy loss, where the weights are the inverse of the normalized classes’ frequency. The pre-training objectives for VICReg and MAE are described respectively in Sections “Self-contrastive learning” and “Self-prediction and generative learning”. The ResNet-34 models pre-trained with SL and VICReg were optimized following the VICReg official implementation^78^, while the ViT-based encoders pre-trained with MAE underwent optimization following its official implementation^79^. Additional details about the optimizer and hyperparameter choices are in Section “Hyperparameters and configuration settings”.

#### Fine-tuning optimization

**Fig. 2:**
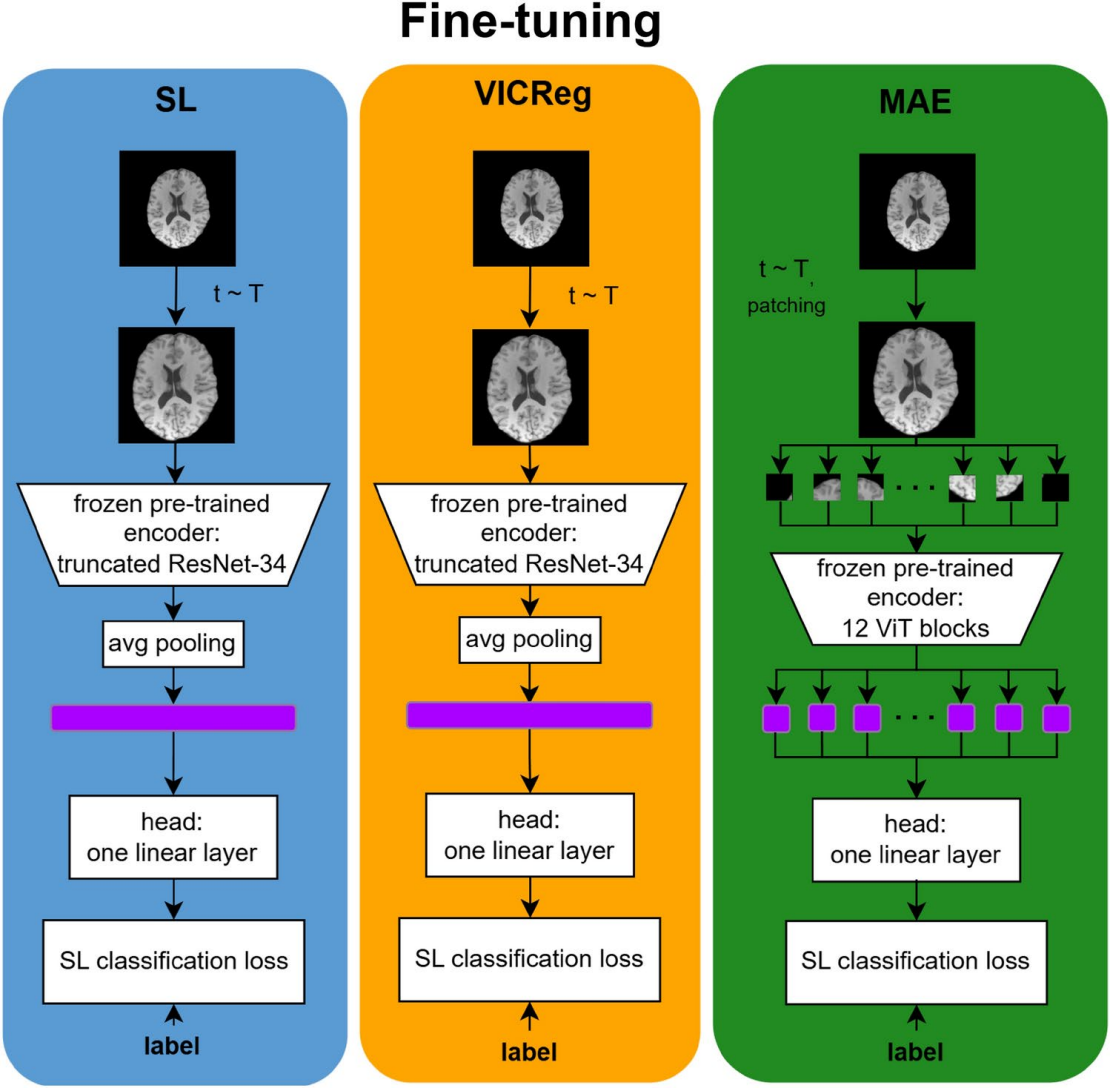
Fine-tuning workflow for the models pre-trained with SL, VICReg, and MAE. For all learning strategies, the encoder is extracted from the pre-trained model, while the projector is replaced with a randomly initialized linear layer (head). The encoder is frozen during the fine-tuning phase. T is the distribution of random transformations for the training set during the fine-tuning phase described in the data augmentation paragraph. A set of transformations t is sampled from T and applied to the batch images. The figure shows brain MRI images as an example, but the same schema applies to chest radiograms or OCT images. (MAE, Masked Autoencoder; MRI, Magnetic Resonance Imaging; OCT, Optical Coherence Tomography; SL, Supervised Learning; VICReg, Variance-Invariance-Covariance Regularization).

For each pre-trained model, the encoder was extracted from the checkpoint corresponding to the epoch with the lowest training loss. The projector or decoder was then replaced with a randomly initialized linear head consisting of 512 input nodes for models based on the ResNet-34 backbone, 384 input nodes for models based on the ViT backbone, and a single output node. The fine-tuning objective for all models was a binary classification task, distinguishing between the following class pairs for each dataset: ‘adults vs. children and adolescents’ (HEALTHY-LIFESPAN meta-dataset), ‘patients with Alzheimer’s disease vs. cognitively normal subjects’ (ADNI meta-dataset), ‘patients with pneumonia vs. healthy controls’ (PneumoniaMNIST dataset), and ‘patients with retinal diseases associated with choroidal neovascularization vs. healthy controls’ (OCTMNIST dataset). We adopted a weighted binary cross-entropy loss, where the weights were the inverse of the normalized classes’ frequency. Figure 2 shows the fine-tuning of the models pre-trained with the different learning strategies.

### Hyperparameters and configuration settings

Systematic hyperparameter tuning is computationally intensive, particularly when working with imaging data. This challenge is even more pronounced in SSL, where the fine-tuning phase is required to access validation metrics on the downstream task. Moreover, when dealing with small datasets, hyperparameter tuning can lead to overfitting the validation set. As a result, it is not uncommon for studies in similar scenarios to set hyperparameters based on previous works rather than conducting systematic tuning^41,80–85^. In our study, we, therefore, initialized the hyperparameters and configuration settings based on the original studies that introduced the paradigms, as well as other applications in comparable scenarios^35,62,76,77,86,87^. We then closely monitored the training process to ensure stability.

Table 1 presents the configuration settings for SL and VICReg, while Table 2 reports those for MAE, which differ due to the models’ distinct backbones. The batch size for pre-training was set to 256 to ensure feasibility with constrained computational resources. During fine-tuning, the model backbone was frozen, allowing the batch size to be increased to 512. The model architecture and batch size were chosen to ensure that both pre-training and fine-tuning required no more than 12 GB of GPU RAM. If a hyperparameter is not explicitly mentioned, it was set to its default value as specified in the original code repository^78,79^.

**Table 1:** Configuration settings for SL and VICReg across all datasets. Since the OCTMNIST dataset is significantly larger than the others, the number of epochs was reduced, as indicated in parentheses.

SL & VICReg configuration	Pre-training value	Fine-tuning value
Optimizer	LARS	SGD
Base learning rate	0.05	0.001
Weight decay	$1.0\times {10}^{-6}$	$1.0\times {10}^{-6}$
Max epochs	1000 (OCTMNIST: 50)	500 (OCTMNIST: 20)
Min epochs	500 (OCTMNIST: 15)	200 (OCTMNIST: 10)
Batch size	256	512
Learning rate schedule	Cosine annealing	Cosine annealing
Warmup epochs	10	20
Early stopping tolerance	0	0
Early stopping patience	150	50 (OCTMNIST:5)
Invariance coefficient $\lambda$ (VICReg)	25	–
Variance coefficient $\mu$ (VICReg)	25	–
Covariance coefficient $\beta$ (VICReg)	1	–

LARS, Layer-wise Adaptive Rate Scaling; SGD, Stochastic Gradient Descent; SL, Supervised Learning; VICReg, Variance-Invariance-Covariance Regularization.

**Table 2:** Configuration settings for MAE.

MAE configuration	Pre-training value	Fine-tuning value
Optimizer	AdamW (0.9, 0.95)	SGD
Base learning rate	$1.5\times {10}^{-4}$	0.001
Weight decay	0.05	$1.0\times {10}^{-6}$
Max epochs	300	80
Min epochs	150	40
Batch size	256	512
Learning rate schedule	Cosine annealing	Cosine annealing
Warmup epochs	20	20
Early stopping tolerance	0	0
Early stopping patience	15	10
Batch size	256	512
Masking ratio	0.75	–
Patch size	16	16
Encoder depth	12	12
Encoder attention heads	6	6
Encoder MLP expansion ratio	4	4
Encoder patch embedding dimension	128	–
Decoder depth	4	–
Decoder attention heads	16	–
Decoder MLP expansion rate	4	–

MAE, Masked Autoencoder; MLP, Multi-Layer Perceptron; SGD, Stochastic Gradient Descent.

### Validation scheme

We compared SL and VICReg, both pre-trained and fine-tuned, across all the meta-datasets. In contrast, the MAE paradigm was evaluated only on the larger OCTMNIST dataset, where it was compared with SL and VICReg. This choice was motivated by the different backbone architectures: ViTs generally underperform compared to CNNs when trained on small datasets due to their weaker inductive biases, specifically in spatial relevance and diverse channel representation^88^.

We performed multiple runs of pre-training and fine-tuning using different random seeds controlling data splitting, random sampling, and stochastic processes of code execution. The number of random seeds was set to 30 for experiments with the smaller meta-datasets (HEALTHY-LIFESPAN, ADNI, and PneumoniaMNIST), and to 5 for the larger meta-dataset OCTMNIST (33,484 training images and 7,872 test set images). This reduction was motivated by the expected lower variability in results and the high computational cost—pre-training a single paradigm on a single seed across all label percentages and class frequency distributions required approximately 10 days on a single NVIDIA A100 GPU. Experiments were conducted for each combination of label availability (100%, 10%, and 5% for all datasets, with an additional 1% for OCTMNIST) and class frequency distribution ([10–90]%, [30–70]%, [50–50]% [70–30]% and [90–10]%). For each experiment, label availability and class frequency distribution were determined by randomly sampling subjects from the training set. In SSL paradigms, the reduced label availability assumption only affected the fine-tuning phase, as pre-training does not require labels. The training and test set sizes remained constant for each dataset across different class frequency combinations within the same random seed.

For the HEALTHY-LIFESPAN and ADNI meta-datasets, we used bootstrap resampling to split the data into training and test sets^89^. In each experiment, the training set was generated by sampling subjects with replacement, ensuring that the total number of subjects in the training set (some of which repeated) remained the same as the entire dataset. The test set for each iteration comprised the subjects not included in the training set (out-of-bag samples). When assuming 100% label availability, for the HEALTHY-LIFESPAN meta-dataset, the training set had a mean of 843 images (minimum 824, maximum 862, standard deviation 12), and the test set had a mean of 617 images (minimum 580, maximum 656, standard deviation 21). For ADNI, the training set had a mean of 771 images (minimum 644, maximum 982, standard deviation 68), and the test set had a mean of 570 images (minimum 406, maximum 740, standard deviation 74).

For the PneumoniaMNIST and OCTMNIST datasets, which are publicly available with predefined training, validation, and test splits, we adopted a different validation scheme to prevent data leakage and assess model performance variability. The original training set was used, with variability introduced through different random samplings to achieve the desired class frequency distribution and label percentage. When assuming 100% label availability, the training set included 1,214 images for PneumoniaMNIST and 33,484 for OCTMNIST. The test set, whose size was kept constant, was obtained by random sampling with replacement from the original validation and test sets. The test set mean size was 752 images (minimum 708, maximum 806, standard deviation 25) for PneumoniaMNIST and 7,872 images (minimum 7,766, maximum 7,986, standard deviation 83) for OCTMNIST.

To ensure an easily interpretable performance evaluation, the test sets of each experiment were balanced by randomly sampling subjects from the majority class to achieve a 50%-50% class distribution. We highlight that, for all datasets, we paid particular attention to preventing data leakage by design. Specifically, within each experiment, all images of a subject were allocated to the same set (either training or test). Additionally, data standardization was applied to both the training and test sets using the mean and standard deviation derived solely from the training set.

### Performance metrics and statistical analysis

To evaluate the performance of each experiment on the test set, we employed the Receiving Operating Characteristics (ROC) and precision-recall (PR) curve for each downstream task. For every paradigm and scenario, we computed the mean value of the area under the ROC curve (ROC-AUC) and the precision-recall curve (PR-AUC) across the experiments with different random seeds. For datasets with 30 experiment repetitions, we also computed the 2.5th and 97.5th percentiles. The ROC-AUC measures a model’s ability to distinguish between classes by plotting the true positive rate (TPR) against the false positive rate (FPR) at varying decision thresholds. It evaluates the overall ranking performance of the model, with an AUC of 1 indicating perfect classification and 0.5 reflecting random guessing. In contrast, the PR-AUC focuses on the trade-off between precision (the proportion of true positive predictions among all positive predictions) and recall (the proportion of correctly identified positives) across thresholds.

To achieve the goals of this study, we conducted a thorough statistical analysis with two primary aims, i.e., to evaluate and compare 1) the performance of SSL vs. SL and 2) the robustness of SSL and SL to class frequency imbalance. For the first aim, we assessed the performance under identical configurations of label availability and class imbalance. For each configuration, i.e., a combination of learning strategy, dataset, label availability assumption, and class frequency distribution of the training set, we performed multiple experiments, each controlled by a different random seed. For all datasets, each configuration was evaluated across 30 experiments (pre-training and fine-tuning), except for OCTMNIST, where only 5 experiments were conducted. For each experiment, we calculated the ROC-AUC and PR-AUC on the test set, which was balanced (50–50% class frequency). We compared the metric scores of the learning strategies using a two-tailed paired t-test when both metric scores were normally distributed or a Wilcoxon signed-rank test otherwise.

For the second aim, for each dataset, learning strategy, and label availability, we compared the metrics scores on the test set across the multiple experiments performed with imbalanced class distributions against those with a balanced training set (50%-50%). We applied a two-tailed paired t-test when metric scores were normally distributed or a Wilcoxon signed-rank test otherwise.

All the p-values were adjusted with Bonferroni correction to control for the inflation of type I error rates related to multiple comparisons.

### Additional details

The source code for the losses, the models’ architectures, the optimizers, and the schedulers were taken from the VICReg repository^78^ and MAE repository^79^ and integrated within a single framework for comparison with SL. The experiments were run on two NVIDIA A100-PCIE-40 GB in approximately 60 days.

### Declaration of generative AI and AI-assisted technologies in the writing process

During the preparation of this work, the authors used ChatGPT to rephrase some sentences. After using this tool/service, the authors reviewed and edited the content as needed and took full responsibility for the content of the publication.

## Results

For experiments with 30 random seeds, Tables S3 and S4 present the results of the normality test on ROC-AUC and PR-AUC distributions, respectively, for each dataset, learning strategy, and configuration.

**Fig. 3:**
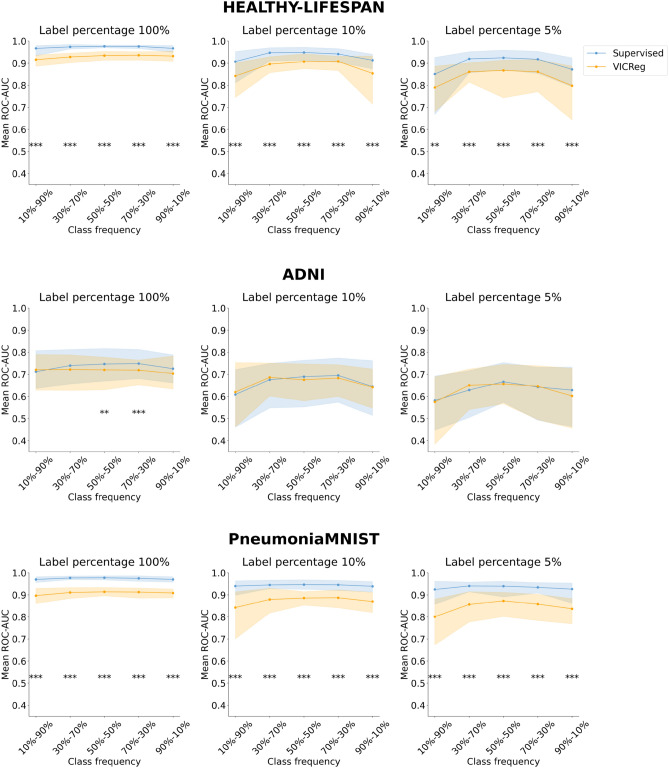
Analysis of the performance of SL and VICReg. Each row corresponds to experiments conducted on a medical imaging dataset. Colored dots represent the mean ROC-AUC scores on the test set across 30 experiments, while shaded regions indicate score variability. The x-axis ticks denote the percentage composition of negative and positive classes in the training set during both pre-training and fine-tuning. The positive class is defined as follows for each dataset: adults and elders (HEALTHY-LIFESPAN), patients diagnosed with Alzheimer’s disease (ADNI), and patients diagnosed with pneumonia (PneumoniaMNIST). For example, an x-tick of 10–90% means that the training set comprised 10% healthy controls and 90% patients diagnosed with a disease (or “children or adolescents” and adults). Results of the statistical comparison between SL and VICReg are summarized using significance symbols: *indicates *p*-value < 0.05, **indicates *p*-value < 0.01, and ***indicates *p*-value < 0.001.

**Fig. 4:**
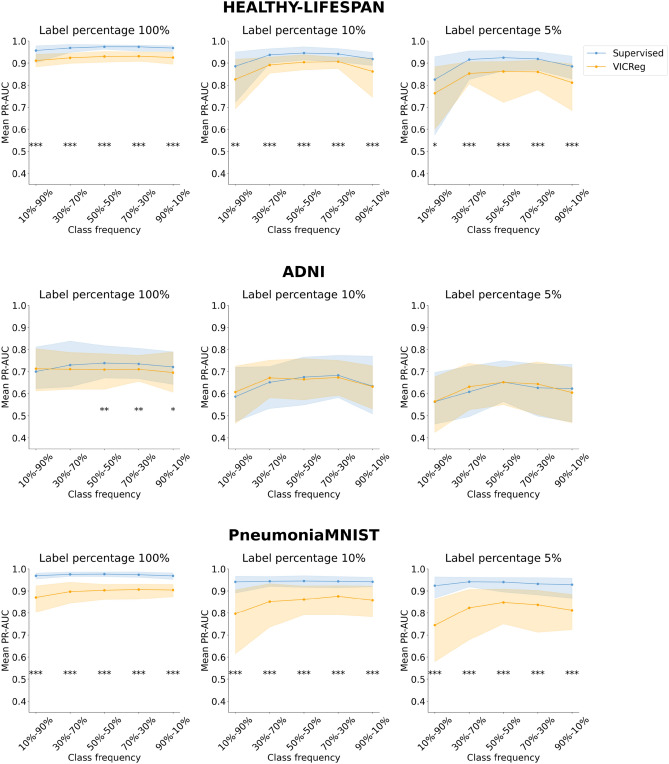
Analysis of the performance of SL and VICReg. Each row corresponds to experiments conducted on a medical imaging dataset. Colored dots represent the mean PR-AUC scores on the test set across 30 experiments, while shaded regions indicate score variability. The x-axis ticks denote the percentage composition of negative and positive classes in the training set during both pre-training and fine-tuning. The positive class is defined as follows for each dataset: adults and elders (HEALTHY-LIFESPAN), patients diagnosed with Alzheimer’s disease (ADNI), and patients diagnosed with pneumonia (PneumoniaMNIST). For example, an x-tick of 10–90% means that the training set comprised 10% healthy controls and 90% patients diagnosed with a disease (or “children or adolescents” and adults). Results of the statistical comparison between SL and VICReg are summarized using significance symbols: *indicates *p*-value < 0.05, **indicates *p*-value < 0.01, and ***indicates *p*-value < 0.001.

**Fig. 5:**
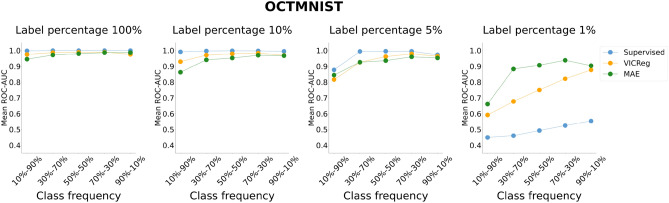
Analysis of the performance of SL, VICReg, and MAE on the OCTMNIST dataset. Colored dots represent the mean ROC-AUC scores on the test set across five experiments, while shaded regions indicate score variability. The x-axis ticks denote the percentage composition of negative and positive classes in the training set during both pre-training and fine-tuning. For example, an x-tick of 10–90% means that the training set comprised 10% healthy controls and 90% patients with choroidal neovascularization.

**Fig. 6:**
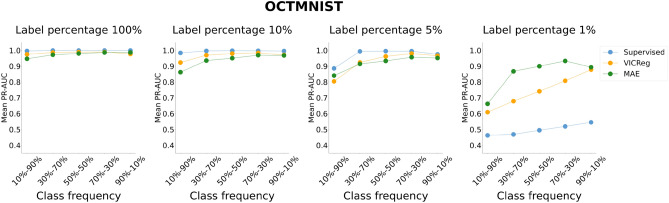
Analysis of the performance of SL, VICReg, and MAE on the OCTMNIST dataset. Colored dots represent the mean PR-AUC scores on the test set across five experiments, while shaded regions indicate score variability. The x-axis ticks denote the percentage composition of negative and positive classes in the training set during both pre-training and fine-tuning. For example, an x-tick of 10–90% means that the training set comprised 10% healthy controls and 90% patients with choroidal neovascularization.

Figures 3, 4, 5, and 6 represent the mean performance and variability (where applicable) of the learning paradigms on the test sets. The colored dots indicate the mean metric score (reported on the y-axis) across multiple experiments with the same configuration varying the random seed. The shaded regions represent the variability in performance, specifically bound by the 2.5th percentile (lower limit of the region) and the 97.5th percentile (upper limit of the region) of the metric scores on the test set across the multiple experiments with the same configuration varying the random seed. The wider the shaded region, the larger the variability of the evaluation metric scores. Detailed results and the statistical analysis results (where applicable) are reported in Tables S5, S6, S7, and S8 for the ROC-AUC and in Tables S9, S10, S11, and S12 for the PR-AUC.

**Fig. 7:**
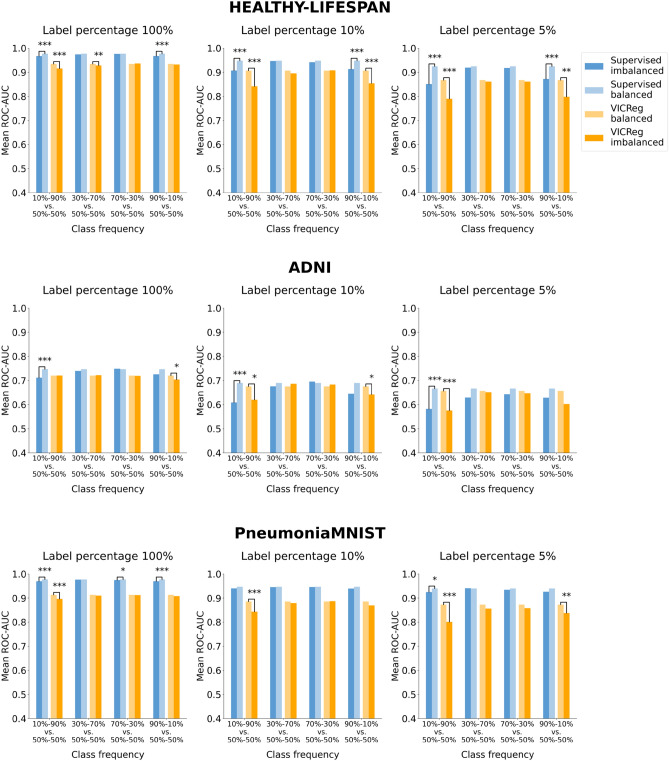
Analysis of the robustness of SL and VICReg to class imbalance. Each row corresponds to experiments conducted on a medical imaging dataset. Each bar represents the mean ROC-AUC score on the test set across 30 experiments. The x-axis ticks denote the percentage composition of negative and positive classes in the training set during both pre-training and fine-tuning. The positive class is defined as follows for each dataset: adults and elders (HEALTHY-LIFESPAN), patients diagnosed with Alzheimer’s disease (ADNI), and patients diagnosed with pneumonia (PneumoniaMNIST). For example, an x-tick of 10–90% means that the training set comprised 10% healthy controls and 90% patients diagnosed with a disease (or “children or adolescents” and adults). Results of the statistical comparison between SL and VICReg are summarized using significance symbols: *indicates *p*-value < 0.05, **indicates *p*-value < 0.01, and ***indicates *p*-value < 0.001.

**Fig. 8:**
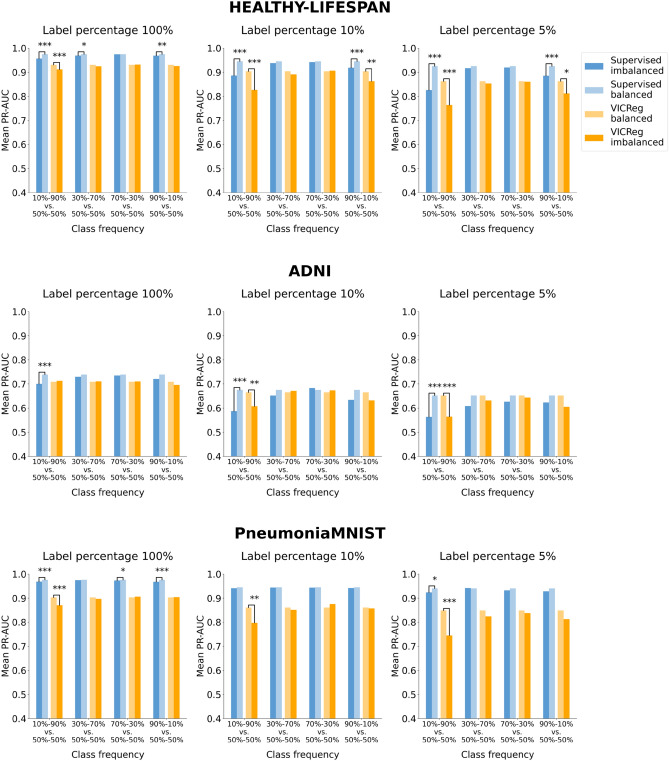
Analysis of the robustness of SL and VICReg to class imbalance. Each row corresponds to experiments conducted on a medical imaging dataset. Each bar represents the mean PR-AUC score on the test set across 30 experiments. The x-axis ticks denote the percentage composition of negative and positive classes in the training set during both pre-training and fine-tuning. The positive class is defined as follows for each dataset: adults and elders (HEALTHY-LIFESPAN), patients diagnosed with Alzheimer’s disease (ADNI), and patients diagnosed with pneumonia (PneumoniaMNIST). For example, an x-tick of 10–90% means that the training set comprised 10% healthy controls and 90% patients diagnosed with a disease (or “children or adolescents” and adults). Results of the statistical comparison between SL and VICReg are summarized using significance symbols: *indicates *p*-value < 0.05, **indicates *p*-value < 0.01, and ***indicates *p*-value < 0.001.

**Fig. 9:**
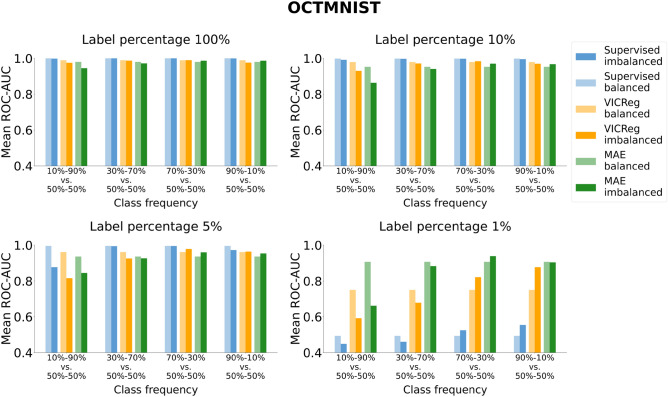
Analysis of the robustness of SL, VICReg, and MAE to class imbalance on the OCTMNIST dataset. Each bar represents the mean ROC-AUC score on the test set across five experiments. The x-axis ticks denote the percentage composition of negative and positive classes in the training set during both pre-training and fine-tuning. For example, an x-tick of 10–90% means that the training set comprised 10% healthy controls and 90% patients with choroidal neovascularization.

**Fig. 10:**
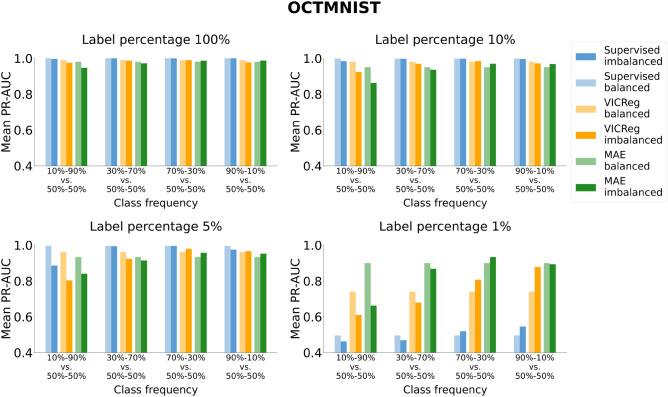
Analysis of the robustness of SL, VICReg, and MAE to class imbalance on the OCTMNIST dataset. Each bar represents the mean PR-AUC score on the test set across five experiments. The x-axis ticks denote the percentage composition of negative and positive classes in the training set during both pre-training and fine-tuning. For example, an x-tick of 10–90% means that the training set comprised 10% healthy controls and 90% patients with choroidal neovascularization.

Figures 7, 8, 9, and 10 illustrate the robustness of the learning paradigms to class imbalance. For each dataset, learning strategy, and level of label availability, we plotted the test set metric scores obtained with imbalanced class distributions and those obtained with a balanced training set (50–50%) to highlight their difference. Each colored bar represents the mean metric value on the test set across repeated experiments. Detailed results and statistical analysis results (where applicable) are reported in Tables S13, S14, S15, and S16 for the ROC-AUC and in Tables S17, S18, S19, and S20 for the PR-AUC.

The findings indicate no significant discrepancies between the ROC-AUC and PR-AUC metrics. In terms of absolute performance, SL generally outperforms SSL across all datasets and settings, except for the OCTMNIST experiments with a 1% label percentage, where SSL surpasses SL by a considerable margin. This performance gap is particularly pronounced for MAE in this configuration when the training dataset is balanced. Additionally, MAE outperforms VICReg when fewer labels are available.

When analyzing robustness to class imbalance, we observe for all paradigms a greater decrease in performance when the training set has a lower frequency of negative class (healthy subjects or “children and adolescent” subjects for the HEALTHY-LIFESPAN meta-dataset), especially for the class frequency 10–90%. Such a strong imbalance causes a sharper drop in SSL performance compared to SL, particularly in ADNI and PneumoniaMNIST datasets. Besides the experiments with the ADNI dataset, SL methods exhibit greater robustness to class imbalance than SSL in most settings.

The results highlight that for both SL and SSL, but particularly for VICReg, the performance varies substantially in repeated runs with different random seeds.

## Discussion

One of the key challenges in medical imaging is the limited availability of large, well-annotated datasets due to privacy policies and data-sharing restrictions. These limitations contribute to the prevalence of small, imbalanced datasets, making the evaluation of SSL methods particularly relevant in such a scenario. In clinical practice, privacy regulations such as GDPR^90^ and HIPAA^91^ impose strict controls on patient data usage, leading to fragmented datasets across institutions. This limited data availability further amplifies the challenge of developing AI models, particularly for SSL techniques that typically rely on large-scale datasets for pre-training to extract meaningful representations. As observed in our experiments, SSL paradigms struggle to achieve competitive performance in 2D medical imaging classification when faced with small datasets and constrained computational resources. The objectives of this study were to evaluate and compare the performance and robustness to class frequency imbalance of SSL vs. SL under identical configurations of label availability and class frequency distribution. Our findings revealed several important insights into the behavior and robustness of these learning paradigms. We emphasize that while the literature has demonstrated the great potential of SSL when large datasets and high-end computational resources are available for pre-training^7^, our study focuses on a setting where only a small dataset for the task of interest is available, and experiments are conducted under constrained computational resources. Across all tasks, the selected SSL paradigms did not outperform SL in terms of absolute performance or robustness to class imbalance, even when label availability was limited. The only exception was observed in the OCTMNIST experiments, where SSL surpassed SL. Notably, OCTMNIST is significantly larger (~ 30 k samples) compared to the other three datasets used in this study (~ 1 k samples), reinforcing the hypothesis that SSL is a preferable choice over SL only when a large partly labeled dataset is available. Importantly, SSL does not require labeled data for pre-training, which distinguishes it from SL and makes it especially appealing in medical imaging, where labels are often difficult or expensive to obtain. In our experiments, the same dataset was used for both SSL pre-training and SL fine-tuning. While this provides a controlled comparison, it does not fully exploit SSL’s strengths. An advantageous scenario would involve using different, large, unlabeled datasets from a similar domain for pre-training—something that SL could not leverage. Therefore, our findings can be seen as a conservative estimate of SSL’s effectiveness. We noticed in our experiments that the largest drops in performance for both SL and SSL were observed when the negative class (healthy subjects for the diagnosis tasks and “children and adolescents” for the age prediction task) was poorly represented. This suggests that the heterogeneity of the negative class might be higher than the heterogeneity among the patients, suggesting the need for more samples to allow the model to learn their variability. This trend is particularly relevant in medical imaging, where acquiring data from healthy subjects is typically easier than obtaining patient data. Therefore, this finding may justify efforts to expand datasets with negative cases. Regarding the robustness to class imbalance, several solutions have been proposed in the literature to improve the performance of SSL, such as reformulating the loss to prefer power-law distributed features^13^, weighting samples inversely correlated with the frequency of their corresponding class using kernel density estimation on the representations even without access to labels^40^, or clustering-based hard negative mining^12^. However, although these approaches might increase the robustness to class imbalance while increasing the workflow complexity and computational resource requirements, they are not solutions to improve the performance in the balanced case. We believe that future works should focus on developing tailored SSL paradigms that exploit prior knowledge about the specific medical dataset of interest rather than relying on general-purpose SSL paradigms to improve simultaneously performance and robustness to class imbalance.

The observed superior performance of SL compared to the selected SSL paradigms in our study may stem from multiple factors, including dataset size and key methodological choices made to ensure a fair comparison between the learning paradigms. Unlike many studies in the literature, our SL models underwent a two-stage training process, with pre-training followed by fine-tuning while replacing the projector, mirroring the training dynamics typically used in SSL. Additionally, to mitigate the impact of class imbalance, SL was equipped with a weighted loss function based on the inverse of class frequency, allowing it to better leverage the available labeled data, which is its main advantage over SSL. Crucially, the SL paradigm was equipped with the same data augmentations as the SSL paradigm. This is a key aspect of ensuring a fair comparison, as it allows SL to operate under conditions that optimize its performance. These design choices likely influenced the observed performance differences, highlighting the importance of methodological rigor in comparative studies of SL and SSL.

Beyond performance and robustness to class imbalance, we emphasize that all paradigms—particularly VICReg—exhibited considerable variability across runs with different random seeds. This underscores the necessity of conducting repeated experiments before drawing any conclusions. The high variability in the performance of SSL, raises concerns about its reliability in clinical settings, where consistency is crucial. Our findings reinforce the importance of methodological rigor and repeated experimentation in evaluating SSL for medical applications.

Additionally, while data augmentations are not essential for SL, they serve as enhancements to improve model performance. In contrast, self-contrastive learning fundamentally depends on these transformations to extract meaningful representations from unlabeled data, making it less suited for scenarios with constrained computational resources compared to SL. SSL methods like VICReg and MAE have been shown in original studies to perform better with larger batch sizes. However, these requirements significantly increase computational demands, potentially limiting the feasibility of SSL implementation in resource-constrained healthcare settings. The poor performance of SSL in the considered scenarios –except for experiments with the larger OCTMNIST dataset—suggests that current SSL paradigms are not well-suited for medical imaging tasks when the training dataset is limited to approximately 1 k samples and computational resources are constrained. However, while our work focused on evaluating SSL in a constrained setting and ensuring a fair comparison with SL, we acknowledge that the observed performance gaps would likely be significantly reduced—or even reversed—when fully leveraging the potential of SSL through pre-training on large unlabeled datasets from similar or even unrelated domains, as demonstrated in previous studies^7,25^.

Although this study is methodological, its findings may have potential implications for clinical practice. By identifying the most effective learning paradigm based on data availability and characteristics, researchers can streamline the development of clinical tools. In the literature, a common approach is to leverage a foundation model pre-trained with SSL on a large dataset before fine-tuning it on the dataset of interest. However, this strategy has notable drawbacks: it can be susceptible to domain shift—especially if a suitable foundation model is unavailable for the specific domain—and is computationally expensive. In this study, we demonstrate that in the considered scenarios, although an AI practitioner might be inclined to use SSL for pre-training on the available portion of data, SL could still be the more effective choice. While acknowledging the immense potential of SSL, we aim to highlight its limitations and encourage research toward more cost-effective and specialized approaches in this field. Specifically, we suggest that SSL could be made more efficient by integrating prior knowledge of the medical data of interest into its learning paradigm.

## Limitations

This study has several limitations. First, we selected hyperparameters based on previous works and did not perform systematic hyperparameter tuning. Since SSL typically involves more hyperparameters than SL, its performance might improve with careful optimization. However, hyperparameter tuning is inherently challenging when working with relatively small datasets. Second, using larger batch sizes or additional data augmentations could potentially enhance SSL performance more than SL. However, implementing either of these approaches is often impractical when computational resources are limited, such as when using a few state-of-the-art high-performance GPUs. Third, our findings may not fully generalize to other imaging modalities, medical tasks, or SSL approaches beyond those evaluated. As performance can vary across tasks, data types, and SSL paradigms, further studies are needed to assess the broader applicability of our conclusions. Fourth, we evaluated SSL using the same dataset for both pre-training and fine-tuning, aiming to test its effectiveness without relying on large unlabeled datasets from related domains, which have been shown in previous studies to improve performance^25^. Finally, we did not evaluate the impact of these methods in a clinical setting, as it was beyond the scope of this study.

## Conclusion

In conclusion, while SSL holds promise for reducing dependency on labeled data, the paradigms evaluated in this study did not outperform SL in terms of performance and robustness to class imbalance when applied to the considered relatively small and imbalanced medical datasets for 2D medical imaging classification tasks. SSL consistently outperformed SL only when a substantially larger training dataset (~ 30k samples) was available; however, its robustness to class imbalance remained limited. These findings emphasize the need for careful evaluation of learning paradigms based on specific application requirements, which depend on factors such as training set size, label availability, and class frequency distribution.

## Supplementary Information

Supplementary Information 1.

Supplementary Information 2.

## Data Availability

The brain MR T_1_-weighted images analyzed during the current study are available in the following online repositories: Autism Brain Imaging Data Exchange (ABIDE), https://fcon_1000.projects.nitrc.org/indi/abide/^63^. Information eXtraction from Images (IXI) study, https://brain-development.org/ixi-dataset^66^. 1000 Functional Connectomes Project (FCP) – ICBM dataset, https://fcon_1000.projects.nitrc.org/fcpClassic/FcpTable.html^67^. Consortium for Reliability and Reproducibility (CoRR) - *NKI 2 - Na*than Kline Institute (Milham), https://fcon_1000.projects.nitrc.org/indi/CoRR/html/index.html^68^. Alzheimer’s Disease Neuroimaging Initiative (ADNI) dataset, https://www.adni.loni.usc.edu/^92^. The chest radiograms (PneumoniaMNIST) and optical coherence tomography (OCTMNIST) images analyzed in this study are available in the MedMNIST + repository at https://www.github.com/MedMNIST/MedMNIST/blob/main/on_medmnist_plus.md^70^.

## Code availability

The source code used for this study is publicly available in a GitHub repository at https://github.com/aiformedresearch/medical-imaging-SSL-vs-SL. The versions of the software, libraries, and toolkits used in our study are listed below Programming language: Python 3.10.13. Python packages: pytorch 2.0.0 = py3.10_cuda11.8_cudnn8.7.0_0, torchvision0.15.0 = py310_cu118, nibabel 5.1.0, torchio 0.19.2, scikit-learn 1.3.0, scipy 1.11.2, numpy 1.25.2. Neuroimaging software: FreeSurfer 7.1.1 for all T1-weighted images except for those belonging to ICBM and NKI2 datasets (FreeSurfer 5.3) and ABIDE I (FreeSurfer 5.1), fractalbrain toolkit 1.0.

## References

[1] Aminizadeh, S. et al. Opportunities and challenges of artificial intelligence and distributed systems to improve the quality of healthcare service. *Artif. Intell. Med.* **149**, 102779. 10.1016/j.artmed.2024.102779 (2024). 38462281 10.1016/j.artmed.2024.102779

[2] Aljuaid, A. & Anwar, M. Survey of supervised learning for medical image processing. *SN Comput. Sci.* **3**, 292. 10.1007/s42979-022-01166-1 (2022). 35602289 10.1007/s42979-022-01166-1 PMC9112642

[3] El Mrabet, M.A., El Makkaoui, K., Faize, A. Supervised machine learning: A Survey. In 2021 4th International Conference on Advanced Communication Technologies and Networking (CommNet), 2021, p. 1–10. 10.1109/CommNet52204.2021.9641998.

[4] Shrestha, A. & Mahmood, A. Review of deep learning algorithms and architectures. *IEEE Access* **7**, 53040–53065. 10.1109/ACCESS.2019.2912200 (2019).

[5] Miotto, R., Wang, F., Wang, S., Jiang, X. & Dudley, J. T. Deep learning for healthcare: Review, opportunities and challenges. *Brief. Bioinform.* **19**, 1236–1246. 10.1093/bib/bbx044 (2018). 28481991 10.1093/bib/bbx044 PMC6455466

[6] Wilkinson, J. et al. Time to reality check the promises of machine learning-powered precision medicine. *The Lancet Digit. Health* **2**, e677–e680. 10.1016/S2589-7500(20)30200-4 (2020). 33328030 10.1016/S2589-7500(20)30200-4 PMC9060421

[7] Azizi, S. et al. Robust and data-efficient generalization of self-supervised machine learning for diagnostic imaging. *Nat. Biomed. Eng.* **7**, 756–779. 10.1038/s41551-023-01049-7 (2023). 37291435 10.1038/s41551-023-01049-7

[8] Rani, V., Nabi, S. T., Kumar, M., Mittal, A. & Kumar, K. Self-supervised learning: A succinct review. *Arch. Computat. Methods Eng.* **30**, 2761–2775. 10.1007/s11831-023-09884-2 (2023). 10.1007/s11831-023-09884-2 PMC9857922 36713767

[9] Gui, J., Chen, T., Zhang, J., Cao, Q., Sun, Z., Luo, H., et al. A survey on self-supervised learning: Algorithms, applications, and future trends. IEEE Transactions on Pattern Analysis and Machine Intelligence 2024:1–20. 10.1109/TPAMI.2024.3415112. 10.1109/TPAMI.2024.3415112 38885108

[10] Deng, J. et al. ImageNet: A large-scale hierarchical image database. *IEEE Conf. Comput. Vision Pattern Recog.* **2009**, 248–255. 10.1109/CVPR.2009.5206848 (2009).

[11] CIFAR-10 and CIFAR-100 n.d. https://www.cs.toronto.edu/~kriz/cifar.html; Accessed July 19, 2024.

[12] Tian, Y., Henaff, O.J., Oord, A. van den. Divide and contrast: Self-supervised learning from uncurated data 2021. 10.48550/arXiv.2105.08054.

[13] Assran, M., Balestriero, R., Duval, Q., Bordes, F., Misra, I., Bojanowski, P., et al. The hidden uniform cluster prior in self-supervised learning 2022. 10.48550/arXiv.2210.07277.

[14] Ren, Z., Wang, S. & Zhang, Y. Weakly supervised machine learning. *CAAI Trans. Intell. Technol.* **8**, 549–580. 10.1049/cit2.12216 (2023).

[15] Shurrab, S. & Duwairi, R. Self-supervised learning methods and applications in medical imaging analysis: A survey. *PeerJ Comput. Sci.* **8**, e1045. 10.7717/peerj-cs.1045 (2022). 36091989 10.7717/peerj-cs.1045 PMC9455147

[16] You, C., Yang, J., Chapiro, J., Duncan, J.S. Unsupervised wasserstein distance guided domain adaptation for 3D multi-domain liver segmentation. In: Cardoso J, Van Nguyen H, Heller N, Henriques Abreu P, Isgum I, Silva W, et al., editors. Interpretable and Annotation-Efficient Learning for Medical Image Computing, Cham: Springer International Publishing; 2020, p. 155–63. 10.1007/978-3-030-61166-8_17.

[17] You, C. et al. Class-aware adversarial transformers for medical image segmentation. *Adv. Neural Inf. Process Syst.* **35**, 29582–29596 (2022). 37533756 PMC10395073

[18] You, C., Zhou, Y., Zhao, R., Staib, L. & Duncan, J. S. SimCVD: Simple contrastive voxel-wise representation distillation for semi-supervised medical image segmentation. *IEEE Trans. Med. Imaging* **41**, 2228–2237. 10.1109/TMI.2022.3161829 (2022). 35320095 10.1109/TMI.2022.3161829 PMC10325835

[19] You, C., Xiang, J., Su, K., Zhang, X., Dong, S., Onofrey, J., et al. Incremental learning meets transfer learning: Application to multi-site prostate MRI segmentation. In: Albarqouni S, Bakas S, Bano S, Cardoso MJ, Khanal B, Landman B, et al., editors. Distributed, Collaborative, and Federated Learning, and Affordable AI and Healthcare for Resource Diverse Global Health, Cham: Springer Nature Switzerland; 2022, p. 3–16. 10.1007/978-3-031-18523-6_1. 10.1007/978-3-031-18523-6_1 PMC10323962 37415747

[20] You, C., Dai, W., Min, Y., Staib, L., Duncan, J.S. Bootstrapping semi-supervised medical image segmentation with anatomical-aware contrastive distillation. In Information Processing in Medical Imaging: 28th International Conference, IPMI 2023, San Carlos de Bariloche, Argentina, June 18–23, 2023, Proceedings, Berlin, Heidelberg: Springer-Verlag; 2023, p. 641–53. 10.1007/978-3-031-34048-2_49. 10.1007/978-3-031-34048-2_49 PMC10322187 37409056

[21] You, C. et al. Mine your own anatomy: Revisiting medical image segmentation with extremely limited labels. *IEEE Trans. Pattern Anal. Mach. Intell.* **46**, 11136–11151. 10.1109/TPAMI.2024.3461321 (2024). 10.1109/TPAMI.2024.3461321 PMC11903367 39269798

[22] You, C., Dai, W., Min, Y., Liu, F., Clifton, D.A., Zhou, S.K., et al. Rethinking semi-supervised medical image segmentation: A variance-reduction perspective. In Proceedings of the 37th International Conference on Neural Information Processing Systems, Red Hook, NY, USA: Curran Associates Inc.; 2024, p. 9984–10021. PMC11136570 38813114

[23] You, C., Dai, W., Min, Y., Staib, L., Sekhon, J., Duncan, J.S. ACTION++: Improving Semi-supervised Medical Image Segmentation with Adaptive Anatomical Contrast. In: Greenspan H, Madabhushi A, Mousavi P, Salcudean S, Duncan J, Syeda-Mahmood T, et al., editors. Medical Image Computing and Computer Assisted Intervention – MICCAI 2023, Cham: Springer Nature Switzerland; 2023, p. 194–205. 10.1007/978-3-031-43901-8_19. 10.1007/978-3-031-43901-8_19 PMC11136572 38813456

[24] You, C., Dai, W., Min, Y., Staib, L., Duncan, J.S. Implicit anatomical rendering for medical image segmentation with stochastic experts. In: Greenspan H, Madabhushi A, Mousavi P, Salcudean S, Duncan J, Syeda-Mahmood T, et al., editors. Medical Image Computing and Computer Assisted Intervention – MICCAI, Springer Nature Switzerland; Cham, 2023, p. 561–71. 10.1007/978-3-031-43898-1_54 PMC11151725 38840671

[25] Wolf, D. et al. Self-supervised pre-training with contrastive and masked autoencoder methods for dealing with small datasets in deep learning for medical imaging. *Sci. Rep.* **13**, 20260. 10.1038/s41598-023-46433-0 (2023). 37985685 10.1038/s41598-023-46433-0 PMC10662445

[26] He, K., Fan, H., Wu, Y., Xie, S., Girshick, R. Momentum contrast for unsupervised visual representation learning. 2020 IEEE/CVF Conference on Computer Vision and Pattern Recognition (CVPR), Seattle, WA, USA: IEEE; 2020, p. 9726–35. 10.1109/CVPR42600.2020.00975.

[27] Caron, M. et al. Unsupervised learning of visual features by contrasting cluster assignments. *Adv. Neural Inf. Process. Syst.* **33**, 9912–9924 (2020).

[28] Grill, J.-B., Strub, F., Altché, F., Tallec, C., Richemond, P.H., Buchatskaya, E., et al. Bootstrap your own latent: A new approach to self-supervised Learning 2020. 10.48550/arXiv.2006.07733.

[29] Tian, K., Jiang, Y., Diao, Q., Lin, C., Wang, L., Yuan, Z. Designing BERT for convolutional networks: Sparse and hierarchical masked modeling. arXivOrg 2023. https://arxiv.org/abs/2301.03580v2; Accessed April 3, 2025.

[30] Yang, X., He, X., Zhao, J., Zhang, Y., Zhang, S., Xie, P. COVID-CT-Dataset: A CT scan dataset about COVID-19 2020. 10.48550/arXiv.2003.13865.

[31] Yang, J. et al. MedMNIST v2 - A large-scale lightweight benchmark for 2D and 3D biomedical image classification. *Sci. Data* **10**, 41. 10.1038/s41597-022-01721-8 (2023). 36658144 10.1038/s41597-022-01721-8 PMC9852451

[32] Azizi, S., Mustafa, B., Ryan, F., Beaver, Z., Freyberg, J., Deaton, J., et al. Big self-supervised models advance medical image classification. 2021 IEEE/CVF International Conference on Computer Vision (ICCV), Montreal, QC, Canada: IEEE; 2021, p. 3458–68. 10.1109/ICCV48922.2021.00346.

[33] Liu, Y. et al. A deep learning system for differential diagnosis of skin diseases. *Nat. Med.* **26**, 900–908. 10.1038/s41591-020-0842-3 (2020). 32424212 10.1038/s41591-020-0842-3

[34] Irvin, J. et al. CheXpert: A large chest radiograph dataset with uncertainty labels and expert comparison. *AAAI* **33**, 590–597. 10.1609/aaai.v33i01.3301590 (2019).

[35] Bardes, A., Ponce, J., LeCun, Y. VICReg: Variance-invariance-covariance regularization for self-supervised learning 2022. 10.48550/arXiv.2105.04906.

[36] Assran, M., Caron, M., Misra, I., Bojanowski, P., Bordes, F., Vincent. P., et al. Masked siamese networks for label-efficient learning 2022. 10.48550/arXiv.2204.07141.

[37] Chen, T., Kornblith, S., Norouzi, M., Hinton, G. A simple framework for contrastive learning of visual representations. In Proceedings of the 37th International Conference on Machine Learning, PMLR; 2020, p. 1597–607.

[38] Chen, X., Fan, H., Girshick, R. & He, K. Improved Baselines with Momentum Contrastive Learning 2020.

[39] Chen, X., He, K. Exploring simple siamese representation learning, 2021, p. 15750–8.

[40] Liu, H., HaoChen, J.Z., Gaidon, A., Ma, T. Self-supervised learning is more robust to dataset imbalance 2022. 10.48550/arXiv.2110.05025.

[41] Zhang, C., Zheng, H. & Gu, Y. Dive into the details of self-supervised learning for medical image analysis. *Med. Image Anal.* **89**, 102879. 10.1016/j.media.2023.102879 (2023). 37453236 10.1016/j.media.2023.102879

[42] Setio, A. A. A. et al. Validation, comparison, and combination of algorithms for automatic detection of pulmonary nodules in computed tomography images: The LUNA16 challenge. *Med. Image Anal.* **42**, 1–13. 10.1016/j.media.2017.06.015 (2017). 28732268 10.1016/j.media.2017.06.015

[43] Zhou, Z., Sodha, V., Pang, J., Gotway, M. B. & Liang, J. Models genesis. *Med. Image Anal.* **67**, 101840. 10.1016/j.media.2020.101840 (2021). 33188996 10.1016/j.media.2020.101840 PMC7726094

[44] Zhou, H.-Y., Lu, C., Yang, S., Han, X., Yu, Y. Preservational learning improves self-supervised medical image models by reconstructing diverse contexts, 2021, p. 3499–509.

[45] Huang, S.-C. et al. Self-supervised learning for medical image classification: A systematic review and implementation guidelines. *Npj Digit. Med.* **6**, 1–16. 10.1038/s41746-023-00811-0 (2023). 37100953 10.1038/s41746-023-00811-0 PMC10131505

[46] Liu, X., Zhang, F., Hou, Z., Mian, L., Wang, Z., Zhang, J., et al. Self-supervised learning: generative or contrastive. IEEE Transactions on Knowledge and Data Engineering 2021, 1–1. 10.1109/TKDE.2021.3090866.

[47] Jing, L., Vincent, P., LeCun, Y., Tian, Y. Understanding dimensional collapse in contrastive self-supervised learning 2022. 10.48550/arXiv.2110.09348 Focus to learn more.

[48] Bachman, P., Hjelm, R.D., Buchwalter, W. Learning representations by maximizing mutual information across views. *Adv. Neural Inf. Process. Syst.,* **32**, 2019.

[49] Henaff, O. Data-efficient image recognition with contrastive predictive coding. In Proceedings of the 37th International Conference on Machine Learning, PMLR; 2020, p. 4182–92.

[50] Misra, I., Maaten, L. van der. Self-supervised learning of pretext-invariant representations, 2020, p. 6707–17.

[51] Tian, Y., Krishnan, D. & Isola, P. Contrastive multiview coding. In *Computer vision – ECCV 2020* (eds Vedaldi, A. et al.) 776–794 (Springer International Publishing, 2020).

[52] Caron, M., Touvron, H., Misra, I., Jégou, H., Mairal, J., Bojanowski, P., et al. Emerging properties in self-supervised vision transformers 2021. 10.48550/arXiv.2104.14294.

[53] Ren, Z., Lan, Q., Zhang, Y. & Wang, S. Exploring simple triplet representation learning. *Comput. Struct. Biotechnol. J.* **23**, 1510–1521. 10.1016/j.csbj.2024.04.004 (2024). 38633386 10.1016/j.csbj.2024.04.004 PMC11021836

[54] Liu, Q. et al. SimTriplet: Simple triplet representation learning with a single GPU. In *medical image computing and computer assisted intervention – MICCAI 2021* (eds de Bruijne, M. et al. et al.) 102–112 (Springer International Publishing, 2021).

[55] Li, G., Togo, R., Ogawa, T., Haseyama, M. Triplet self-supervised learning for gastritis detection with scarce annotations. In 2021 IEEE 10th Global Conference on Consumer Electronics (GCCE), 2021, p. 787–8. 10.1109/GCCE53005.2021.9622100.

[56] Zbontar, J., Jing, L., Misra, I., LeCun, Y., Deny, S. Barlow twins: Self-supervised learning via redundancy reduction 2021. 10.48550/arXiv.2103.03230.

[57] Ren, Z., Zhang, Y. & Wang, S. A hybrid framework for lung cancer classification. *Electronics (Basel)* **11**, 1614. 10.3390/electronics1010000 (2022). 36568860 10.3390/electronics1010000 PMC7613986

[58] Auto-GAN: Self-Supervised Collaborative Learning for Medical Image Synthesis | Request PDF. ResearchGate 2024. 10.1609/aaai.v34i07.6619.

[59] Park, I., Kim, S., Ryu, J. Generative self-supervised learning for medical image classification, 2024, 976–93.

[60] Devlin, J., Chang, M.-W., Lee, K., Toutanova, K. BERT: Pre-training of deep bidirectional transformers for language understanding. arXivOrg 2018. https://arxiv.org/abs/1810.04805v2; Accessed January 24, 2025.

[61] Bao, H., Dong, L., Piao, S., Wei, F. BEiT: BERT Pre-Training of Image Transformers. arXivOrg 2021. https://arxiv.org/abs/2106.08254v2; Accessed January 24, 2025.

[62] He, K., Chen, X., Xie, S., Li, Y., Dollár, P., Girshick, R. Masked autoencoders are scalable vision learners. arXivOrg 2021. https://arxiv.org/abs/2111.06377v3; Accessed January 24, 2025.

[63] ABIDE 2024. https://fcon_1000.projects.nitrc.org/indi/abide; Accessed April 23, 2024.

[64] Di Martino, A. et al. Enhancing studies of the connectome in autism using the autism brain imaging data exchange II. *Sci. Data* **4**, 170010. 10.1038/sdata.2017.10 (2017). 28291247 10.1038/sdata.2017.10 PMC5349246

[65] Di Martino, A. et al. The autism brain imaging data exchange: Towards a large-scale evaluation of the intrinsic brain architecture in autism. *Mol. Psychiatry* **19**, 659–667. 10.1038/mp.2013.78 (2014). 23774715 10.1038/mp.2013.78 PMC4162310

[66] IXI Dataset. IXI Dataset – Brain Development 2024. https://www.brain-development.org/ixi-dataset/; Accessed April 23, 2024.

[67] Functional Connectomes Project 2024. https://fcon_1000.projects.nitrc.org/fcpClassic/FcpTable.html; Accessed April 23, 2024.

[68] Consortium for Reliability and Reproducibility (CoRR) 2024. https://www.fcon_1000.projects.nitrc.org/indi/CoRR/html/index.html; Accessed April 23, 2024.

[69] Autism Brain Imaging Data Exchange (ABIDE) 2017. https://fcon_1000.projects.nitrc.org/indi/CoRR/html/.

[70] MedMNIST/on_medmnist_plus.md at main · MedMNIST/MedMNIST. GitHub n.d. https://www.github.com/MedMNIST/MedMNIST/blob/main/on_medmnist_plus.md; Accessed June 25, 2024.

[71] Kermany, D., Zhang, K., Goldbaum, M. Large dataset of labeled optical coherence Tomography (OCT) and chest X-ray images 2018;3. 10.17632/rscbjbr9sj.3.

[72] Jenkinson, M., Beckmann, C. F., Behrens, T. E. J., Woolrich, M. W. & Smith, S. M. FSL. *Neuroimage* **62**, 782–790. 10.1016/j.neuroimage.2011.09.015 (2012). 21979382 10.1016/j.neuroimage.2011.09.015

[73] Fischl, B. FreeSurfer. *Neuroimage* **62**, 774–781. 10.1016/j.neuroimage.2012.01.021 (2012). 22248573 10.1016/j.neuroimage.2012.01.021 PMC3685476

[74] Poloni, K. M. & Ferrari, R. J. A deep ensemble hippocampal CNN model for brain age estimation applied to Alzheimer’s diagnosis. *Expert Syst. Appl.* **195**, 116622. 10.1016/j.eswa.2022.116622 (2022).

[75] Hong, J. et al. Brain age prediction of children using routine brain MR images via deep learning. *Front. Neurol.* **11**, 584682. 10.3389/fneur.2020.584682 (2020). 33193046 10.3389/fneur.2020.584682 PMC7604456

[76] He K, Zhang X, Ren S, Sun J. Deep Residual Learning for Image Recognition. 2016 IEEE Conference on Computer Vision and Pattern Recognition (CVPR), Las Vegas, NV, USA: IEEE; 2016, p. 770–8. 10.1109/CVPR.2016.90.

[77] Dosovitskiy, A., Beyer, L., Kolesnikov, A., Weissenborn, D., Zhai, X., Unterthiner, T., et al. An image is worth 16x16 Words: Transformers for image recognition at scale 2021. 10.48550/arXiv.2010.11929.

[78] facebookresearch/vicreg 2024.

[79] facebookresearch/mae 2025.

[80] Torabi, M., Rasouli, A. H., Wu, Q. M. J., Cao, W. & Pourpanah, F. Self-supervised adversarial adaptation network for breast cancer detection. *Eng. Appl. Artif. Intell.* **133**, 108489. 10.1016/j.engappai.2024.108489 (2024).

[81] Wang, X., Yang, S., Zhang, J., Wang, M., Zhang, J., Huang, J., et al. TransPath: Transformer-based self-supervised learning for histopathological image classification. In: de Bruijne M, Cattin PC, Cotin S, Padoy N, Speidel S, Zheng Y, et al., editors. Medical image computing and computer assisted intervention – MICCAI 2021, Springer International Publishing, 2021, p. 186–95. 10.1007/978-3-030-87237-3_18.

[82] Azizi, S. et al. Big self-supervised models advance medical image classification. *IEEE/CVF Int. Conf. Comput. Vision (ICCV)* **2021**, 3458–3468. 10.1109/ICCV48922.2021.00346 (2021).

[83] Li, X. et al. Rotation-oriented collaborative self-supervised learning for retinal disease diagnosis. *IEEE Trans. Med. Imaging* **40**, 2284–2294. 10.1109/TMI.2021.3075244 (2021). 33891550 10.1109/TMI.2021.3075244

[84] Huang, S.-C. et al. Self-supervised learning for medical image classification: a systematic review and implementation guidelines. *Npj. Digit. Med.* **6**, 1–16. 10.1038/s41746-023-00811-0 (2023). 10.1038/s41746-023-00811-0 PMC10131505 37100953

[85] Bundele, V., Çal, O.A., Kargi, B., Sarıtaş, K., Tezören, K., Ghaderi, Z., et al. Evaluating self-supervised learning in medical imaging: A benchmark for robustness, generalizability, and multi-domain impact 2024. 10.48550/arXiv.2412.19124.

[86] Tan, J.H. Pre-training of lightweight vision transformers on small datasets with minimally scaled images 2024. 10.48550/arXiv.2402.03752.

[87] Touvron, H., Cord, M., Douze, M., Massa, F., Sablayrolles, A., Jégou, H. Training data-efficient image transformers & distillation through attention 2021. 10.48550/arXiv.2012.12877.

[88] Lu, Z., Xie, H., Liu, C., Zhang, Y. Bridging the gap between vision transformers and convolutional neural networks on small datasets, 2022.

[89] Chernick, M.R., González-Manteiga, W., Crujeiras, R.M., Barrios, E.B. Bootstrap Methods. In: Lovric M, editor. International Encyclopedia of Statistical Science, Berlin, Heidelberg: Springer; 2011, p. 169–74. 10.1007/978-3-642-04898-2_150.

[90] General Data Protection Regulation (GDPR) – Legal Text. General Data Protection Regulation (GDPR) n.d. https://gdpr-info.eu/; Accessed January 31, 2025.

[91] Edemekong, P. F., Annamaraju, P., Afzal, M. & Haydel, M. J. *Health Insurance Portability and Accountability Act (HIPAA) Compliance* (StatPearls Publishing, 2025). 29763195

[92] ADNI. ADNI | Alzheimer’s Disease Neuroimaging Initiative 2024. https://adni.loni.usc.edu/; Accessed July 18, 2024.

