# Supplementary material for: Comparative analysis of supervised and self-supervised learning with small and imbalanced medical imaging datasets
Source: Sci Rep. 2025 Sep 2;15:32345. doi: 10.1038/s41598-025-99000-0 (PMC12405560; doi:10.1038/s41598-025-99000-0)
Supplement: Supplementary file 2 — Supplementary Information 2. [file 41598_2025_99000_MOESM2_ESM.docx]

**Supplemental tables for “Comparative analysis of supervised and self-supervised learning with small and imbalanced medical datasets”**

**Table S1.** Scanning parameters for each center of the HEALTHY-LIFESPAN meta-dataset.

| **Dataset** | **Manufacturer and model** | **Magnetostatic field (T)** | **In-plane resolution**  **(mm** × **mm)** | **Slice thickness (mm)** | **TR (ms)** | **TE (ms)** | **TI (ms)** | **FA (°)** |
| --- | --- | --- | --- | --- | --- | --- | --- | --- |
| ABIDEI-CALTECH | Siemens MAGNETOM Trio | 3 | 1.0 × 1.0 | 1.0 | 1590 | 2.73 | 800 | 10 |
| ABIDEI-CMU | Siemens MAGNETOM Verio | 3 | 1.0 × 1.0 | 1.0 | 1870 | 2.48 | 1100 | 8 |
| ABIDEI-KKI | Philips Achieva | 3 | 1.0 × 1.0 | 1.0 | 8.0 | 3.7 | 843 | 8 |
| ABIDEI-LEUVEN | Philips Intera | 3 | 0.98 × 0.98 | 1.20 | 9.6 | 4.6 | 885 | 8 |
| ABIDEI-MAX_MUN | Siemens MAGNETOM Verio | 3 | 1.0 × 1.0 | 1.0 | 1800 | 3.06 | 900 | 9 |
| ABIDEI-NYU | Siemens MAGNETOM Allegra | 3 | 1.3 × 1.0 | 1.3 | 2530 | 3.25 | 1100 | 7 |
| ABIDEI-OHSU | Siemens MAGNETOM Trio | 3 | 1.0 × 1.0 | 1.1 | 2300 | 3.58 | 900 | 10 |
| ABIDEI-OLIN | Siemens MAGNETOM Allegra | 3 | 1.0 × 1.0 | 1.0 | 2500 | 2.74 | 900 | 8 |
| ABIDEI-PITT | Siemens MAGNETOM Allegra | 3 | 1.1 × 1.1 | 1.1 | 2100 | 3.93 | 1000 | 7 |
| ABIDEI-SBL | Philips Intera | 3 | 1.0 × 1.0 | 1.0 | 9.0 | 3.5 | 144 | 8 |
| ABIDEI-SDSU | GE Discovery MR750 | 3 | 1.0 × 1.0 | 1.0 | 11.08 | 4.3 | 600 | 45 |
| ABIDEI-STANFORD | GE Signa | 3 | 0.859 × 1.500 | 0.859 | 8.4 | 1.8 | NA | 15 |
| ABIDEI-TRINITY | Philips Achieva | 3 | 1.0 × 1.0 | 1.0 | 8.5 | 3.9 | 1060 | 8 |
| ABIDEI-UCLA | Siemens MAGNETOM Trio | 3 | 1.0 × 1.0 | 1.2 | 2300 | 2.84 | 853 | 9 |
| ABIDEI-UM | GE Signa | 3 | NA | 1.2 | NA | 1.8 | NA | 15 |
| ABIDEI-USM | Siemens MAGNETOM Trio | 3 | 1.0 × 1.0 | 1.2 | 2300 | 2.91 | 900 | 9 |
| ABIDEII-BNI_1 | Philips Ingenia | 3 | 1.1 × 1.1 | 1.2 | 6.7 | 3.1 | 799 | 9 |
| ABIDEII-EMC_1 | GE Discovery MR750 | 3 | 1.1 × 1.1 | 1.2 | 6.7 | 3.1 | 350 | 9 |
| ABIDEII-ETH_1 | Philips Achieva | 3 | 0.9 × 0.9 | 0.9 | 8.4 | 3.9 | 1150 | 8 |
| ABIDEII-GU_1 | Siemens MAGNETOM Trio | 3 | 1.0 × 1.0 | 1.0 | 2530 | 3.5 | 1100 | 7 |
| ABIDEII-IP_1 | Philips Achieva | 1.5 | 1.0 × 1.0 | 1.0 | 25 | 5.6 | NA | 30 |
| ABIDEII-IU_1 | Siemens TrioTim | 3 | 0.7 × 0.7 | 0.7 | 2400 | 2.3 | 1000 | 8 |
| ABIDEII-KKI_32ch | Philips Achieva | 3 | 0.95 × 0.96 | 1.0 | 8.2 | 3.7 | 753 | 8 |
| ABIDEII-KKI_8ch | Philips Achieva | 3 | 1.0 × 1.0 | 1.0 | 8.0 | 3.7 | 843 | 8 |
| ABIDEII-NYU_1 | Siemens Allegra | 3 | 1.3 × 1.0 | 1.33 | 2530 | 3.25 | 1100 | 7 |
| ABIDEII-OHSU_1 | Siemens TrioTim | 3 | 1.0 × 1.0 | 1.1 | 2300 | 3.58 | 900 | 10 |
| ABIDEII-SDSU_1 | GE Discovery MR750 | 3 | 1.0 × 1.0 | 1.0 | 8.136 | 3.172 | 600 | 8 |
| ABIDEII-TCD_1 | Philips Intera Achieva | 3 | 0.9 × 0.9 | 0.9 | 8.4 | 3.9 | 1150 | 8 |
| ABIDEII-UCD_1 | Siemens TrioTim | 3 | 1.0 × 1.0 | 1.0 | 2000 | 3.16 | 1050 | 8 |
| ABIDEII-UCLA_1 | Siemens TrioTim | 3 | 1.0 × 1.0 | 1.2 | 2300 | 2.86 | 853 | 9 |
| ABIDEII-USM_1 | Siemens TrioTim | 3 | 1.0 × 1.0 | 1.2 | 2300 | 2.91 | 900 | 9 |
| ICBM | NA | 3 | 1.0 × 1.0 | 1.0 | NA | NA | NA | NA |
| IXI-Guys | Philips Gyroscan Intera | 1.5 | NA | NA | 9.813 | 4.603 | NA | 8 |
| IXI-HH | Philips Intera | 3 | NA | NA | 9.6 | 4.6 | NA | 8 |
| IXI-IOP | NA | NA | NA | NA | NA | NA | NA | NA |
| NKI2 | NA | 3 | 1.0 × 1.0 | 1.0 | NA | NA | NA | NA |

FA, flip angle; NA, not available; TE, echo time; TI, inversion time; TR, ­­­­­­­­­­­repetition time

**Table S2.** Description of the demographic characteristics of each center of the HEALTHY-LIFESPAN meta-dataset.

| **Dataset** | **Institution** | **# participants** | **Females (%)** | **Age range**  **min-max** | **Age median (IQR)** |
| --- | --- | --- | --- | --- | --- |
| ABIDEI-CALTECH | California Institute of Technology | 19 | 21.05 | 17.0 - 56.2 | 23.6 (15.85) |
| ABIDEI-CMU | Carnegie Mellon  University | 13 | 23.08 | 20.0 - 40.0 | 27.0 (9.0) |
| ABIDEI-KKI | Kennedy Krieger Institute | 33 | 27.27 | 8.07 - 12.77 | 9.97 (1.54) |
| ABIDEI-LEUVEN | University of Leuven | 35 | 14.29 | 12.2 - 29.0 | 16.6 (7.95) |
| ABIDEI-MAX_MUN | Ludwig Maximilians  University Munich | 33 | 12.12 | 7.0 - 48.0 | 26.0 (9.0) |
| ABIDEI-NYU | New York University Langone Medical Center | 105 | 24.76 | 6.47 - 31.78 | 14.38 (8.99) |
| ABIDEI-OHSU | Oregon Health & Science University | 15 | 0 | 8.2 - 11.99 | 10.08 (1.31) |
| ABIDEI-OLIN | Olin Center, Institute of Living at Hartford Hospital | 16 | 12.5 | 10.0 - 23.0 | 16.5 (6.25) |
| ABIDEI-PITT | University of Pittsburgh School of Medicine | 27 | 14.81 | 9.44 - 33.24 | 17.13 (8.3) |
| ABIDEI-SBL | Social Brain Lab  BCN NeuroImaging Center, University Medical Center Groningen and Netherlands Institute for Neuroscience | 15 | 0 | 20.0 - 42.0 | 36.0 (11.5) |
| ABIDEI-SDSU | San Diego State University | 22 | 27.27 | 8.67 - 16.88 | 14.42 (2.45) |
| ABIDEI-STANFORD | Stanford University | 20 | 20 | 7.75 - 12.43 | 9.41 (2.57) |
| ABIDEI-TRINITY | Trinity College Dublin | 25 | 0 | 12.04 - 25.66 | 15.91 (5.25) |
| ABIDEI-UCLA | University of California,  Los Angeles | 47 | 12.77 | 9.21 - 17.79 | 12.68 (2.16) |
| ABIDEI-UM | University of Michigan | 77 | 23.38 | 8.2 - 28.8 | 14.8 (5.0) |
| ABIDEI-USM | University of Utah  School of Medicine | 43 | 0 | 8.77 - 39.39 | 19.76 (10.4) |
| ABIDEII-BNI_1 | Barrow Neurological Institute | 29 | 0 | 18.0 - 64.0 | 43.0 (27.0) |
| ABIDEII-EMC_1 | Erasmus University Medical Centre | 27 | 18.52 | 6.33 - 10.12 | 8.19 (1.41) |
| ABIDEII-ETH_1 | ETH Zürich | 24 | 0 | 13.83 - 30.67 | 24.0 (6.79) |
| ABIDEII-GU_1 | Georgetown University | 55 | 49.09 | 8.06 - 13.8 | 10.43 (2.96) |
| ABIDEII-IP_1 | Institut Pasteur and Robert Debré Hospital | 34 | 64.71 | 8.07 - 46.6 | 22.12 (18.8) |
| ABIDEII-IU_1 | Indiana University | 20 | 25 | 19.0 - 37.0 | 22.0 (4.25) |
| ABIDEII-KKI_32ch | Kennedy Krieger Institute | 45 | 26.67 | 8.06 - 12.67 | 10.27 (1.67) |
| ABIDEII-KKI_8ch | Kennedy Krieger Institute | 110 | 40 | 8.02 - 12.9 | 10.3 (1.68) |
| ABIDEII-NYU_1 | New York University Langone Medical Center | 30 | 6.67 | 5.89 - 23.81 | 9.11 (3.12) |
| ABIDEII-OHSU_1 | Oregon Health & Science University | 56 | 51.79 | 8.0 - 14.0 | 10.0 (2.25) |
| ABIDEII-SDSU_1 | San Diego State University | 25 | 8 | 8.1 - 17.7 | 13.0 (5.2) |
| ABIDEII-TCD_1 | Trinity College Dublin | 21 | 0 | 10.25 - 20.0 | 15.25 (5.25) |
| ABIDEII-UCD_1 | University of California Davis | 14 | 28.57 | 12.25 - 17.17 | 14.75 (2.65) |
| ABIDEII-UCLA_1 | University of California,  Los Angeles | 16 | 31.25 | 7.76 - 14.09 | 9.01 (1.43) |
| ABIDEII-USM_1 | University of Utah  School of Medicine | 16 | 18.75 | 11.5 - 36.15 | 23.78 (10.72) |
| ICBM | International Consortium for Human Brain Mapping | 86 | 52.33 | 19.0 - 85.0 | 44.5 (31.5) |
| IXI-Guys | Guy’s Hospital - London | 313 | 55.59 | 20.07 - 86.2 | 53.41 (26.08) |
| IXI-HH | Hammersmith Hospital - London | 181 | 51.38 | 20.17 - 81.94 | 48.05 (28.25) |
| IXI-IOP | Institute of Psychiatry - London | 67 | 65.67 | 19.98 - 86.32 | 36.16 (19.85) |
| NKI2 |  | 73 | 41.1 | 6.0 - 17.0 | 12.0 (6.0) |

IQR: interquartile range

**Table S3.** For each configuration of label percentage, meta-dataset, class frequency, and learning strategy, 30 experiments have been carried out. We tested if the ROC-AUC scores of the 30 experiments under the same configuration were normally distributed using the Shapiro-Wilk test. P-values were adjusted using the Bonferroni correction. If the adjusted p-value < 0.05, we considered the ROC-AUC scores not normally distributed.

| **meta-dataset** | **class**  **frequency** | **learning strategy** | **Shapiro-Wilk test p-value** | **Shapiro-Wilk test adjusted p-value** | **deviates from normality** |
| --- | --- | --- | --- | --- | --- |
| **label percentage 100%** | | | | | |
| HEALTHY-LIFESPAN | 10%-90% | Supervised | 1.503e-05 | 7.514e-05 | True |
| HEALTHY-LIFESPAN | 10%-90% | VICReg | 2.729e-01 | 1 | False |
| HEALTHY-LIFESPAN | 30%-70% | Supervised | 5.675e-01 | 1 | False |
| HEALTHY-LIFESPAN | 30%-70% | VICReg | 2.686e-01 | 1 | False |
| HEALTHY-LIFESPAN | 50%-50% | Supervised | 1.305e-01 | 6.526e-01 | False |
| HEALTHY-LIFESPAN | 50%-50% | VICReg | 5.807e-01 | 1 | False |
| HEALTHY-LIFESPAN | 70%-30% | Supervised | 8.928e-03 | 4.464e-02 | True |
| HEALTHY-LIFESPAN | 70%-30% | VICReg | 6.703e-02 | 3.351e-01 | False |
| HEALTHY-LIFESPAN | 90%-10% | Supervised | 2.304e-01 | 1 | False |
| HEALTHY-LIFESPAN | 90%-10% | VICReg | 9.899e-01 | 1 | False |
| **label percentage 10%** | | | | | |
| HEALTHY-LIFESPAN | 10%-90% | Supervised | 3.190e-03 | 1.595e-02 | True |
| HEALTHY-LIFESPAN | 10%-90% | VICReg | 1.619e-02 | 8.096e-02 | False |
| HEALTHY-LIFESPAN | 30%-70% | Supervised | 8.373e-02 | 4.187e-01 | False |
| HEALTHY-LIFESPAN | 30%-70% | VICReg | 9.148e-01 | 1 | False |
| HEALTHY-LIFESPAN | 50%-50% | Supervised | 3.794e-02 | 1.897e-01 | False |
| HEALTHY-LIFESPAN | 50%-50% | VICReg | 7.954e-01 | 1 | False |
| HEALTHY-LIFESPAN | 70%-30% | Supervised | 5.642e-02 | 2.821e-01 | False |
| HEALTHY-LIFESPAN | 70%-30% | VICReg | 9.913e-03 | 4.956e-02 | True |
| HEALTHY-LIFESPAN | 90%-10% | Supervised | 3.005e-01 | 1 | False |
| HEALTHY-LIFESPAN | 90%-10% | VICReg | 9.776e-03 | 4.888e-02 | True |
| **label percentage 5%** | | | | | |
| HEALTHY-LIFESPAN | 10%-90% | Supervised | 2.076e-04 | 1.038e-03 | True |
| HEALTHY-LIFESPAN | 10%-90% | VICReg | 6.018e-01 | 1 | False |
| HEALTHY-LIFESPAN | 30%-70% | Supervised | 1.806e-03 | 9.029e-03 | True |
| HEALTHY-LIFESPAN | 30%-70% | VICReg | 6.153e-01 | 1 | False |
| HEALTHY-LIFESPAN | 50%-50% | Supervised | 3.653e-02 | 1.827e-01 | False |
| HEALTHY-LIFESPAN | 50%-50% | VICReg | 1.680e-04 | 8.401e-04 | True |
| HEALTHY-LIFESPAN | 70%-30% | Supervised | 9.769e-03 | 4.885e-02 | True |
| HEALTHY-LIFESPAN | 70%-30% | VICReg | 1.810e-01 | 9.049e-01 | False |
| HEALTHY-LIFESPAN | 90%-10% | Supervised | 1.330e-02 | 6.651e-02 | False |
| HEALTHY-LIFESPAN | 90%-10% | VICReg | 4.813e-02 | 2.407e-01 | False |
| **label percentage 100%** | | | | | |
| ADNI | 10%-90% | Supervised | 6.899e-01 | 1 | False |
| ADNI | 10%-90% | VICReg | 2.460e-01 | 1 | False |
| ADNI | 30%-70% | Supervised | 4.134e-01 | 1 | False |
| ADNI | 30%-70% | VICReg | 1.761e-02 | 8.804e-02 | False |
| ADNI | 50%-50% | Supervised | 1.172e-01 | 5.861e-01 | False |
| ADNI | 50%-50% | VICReg | 3.770e-01 | 1 | False |
| ADNI | 70%-30% | Supervised | 8.090e-02 | 4.045e-01 | False |
| ADNI | 70%-30% | VICReg | 2.045e-01 | 1 | False |
| ADNI | 90%-10% | Supervised | 9.302e-01 | 1 | False |
| ADNI | 90%-10% | VICReg | 9.562e-01 | 1 | False |
| **label percentage 10%** | | | | | |
| ADNI | 10%-90% | Supervised | 6.863e-01 | 1 | False |
| ADNI | 10%-90% | VICReg | 9.031e-01 | 1 | False |
| ADNI | 30%-70% | Supervised | 9.475e-02 | 4.737e-01 | False |
| ADNI | 30%-70% | VICReg | 1.947e-01 | 9.735e-01 | False |
| ADNI | 50%-50% | Supervised | 4.500e-03 | 2.250e-02 | True |
| ADNI | 50%-50% | VICReg | 5.267e-01 | 1 | False |
| ADNI | 70%-30% | Supervised | 3.152e-01 | 1 | False |
| ADNI | 70%-30% | VICReg | 9.118e-02 | 4.559e-01 | False |
| ADNI | 90%-10% | Supervised | 2.721e-01 | 1 | False |
| ADNI | 90%-10% | VICReg | 5.695e-01 | 1 | False |
| **label percentage 5%** | | | | | |
| ADNI | 10%-90% | Supervised | 1.456e-01 | 7.279e-01 | False |
| ADNI | 10%-90% | VICReg | 3.167e-02 | 1.584e-01 | False |
| ADNI | 30%-70% | Supervised | 4.618e-01 | 1 | False |
| ADNI | 30%-70% | VICReg | 2.046e-01 | 1 | False |
| ADNI | 50%-50% | Supervised | 2.088e-01 | 1 | False |
| ADNI | 50%-50% | VICReg | 9.894e-01 | 1 | False |
| ADNI | 70%-30% | Supervised | 3.636e-02 | 1.818e-01 | False |
| ADNI | 70%-30% | VICReg | 1.027e-01 | 5.137e-01 | False |
| ADNI | 90%-10% | Supervised | 9.672e-02 | 4.836e-01 | False |
| ADNI | 90%-10% | VICReg | 2.787e-01 | 1 | False |
| **label percentage 100%** | | | | | |
| PneumoniaMNIST | 10%-90% | Supervised | 6.862e-01 | 1 | False |
| PneumoniaMNIST | 10%-90% | VICReg | 6.340e-01 | 1 | False |
| PneumoniaMNIST | 30%-70% | Supervised | 5.992e-01 | 1 | False |
| PneumoniaMNIST | 30%-70% | VICReg | 4.105e-01 | 1 | False |
| PneumoniaMNIST | 50%-50% | Supervised | 5.834e-01 | 1 | False |
| PneumoniaMNIST | 50%-50% | VICReg | 5.532e-01 | 1 | False |
| PneumoniaMNIST | 70%-30% | Supervised | 6.013e-01 | 1 | False |
| PneumoniaMNIST | 70%-30% | VICReg | 6.423e-01 | 1 | False |
| PneumoniaMNIST | 90%-10% | Supervised | 8.808e-01 | 1 | False |
| PneumoniaMNIST | 90%-10% | VICReg | 5.511e-01 | 1 | False |
| **label percentage 10%** | | | | | |
| PneumoniaMNIST | 10%-90% | Supervised | 1.872e-05 | 9.359e-05 | True |
| PneumoniaMNIST | 10%-90% | VICReg | 2.236e-03 | 1.118e-02 | True |
| PneumoniaMNIST | 30%-70% | Supervised | 5.829e-01 | 1 | False |
| PneumoniaMNIST | 30%-70% | VICReg | 7.577e-01 | 1 | False |
| PneumoniaMNIST | 50%-50% | Supervised | 9.264e-01 | 1 | False |
| PneumoniaMNIST | 50%-50% | VICReg | 3.737e-01 | 1 | False |
| PneumoniaMNIST | 70%-30% | Supervised | 2.667e-01 | 1 | False |
| PneumoniaMNIST | 70%-30% | VICReg | 4.269e-01 | 1 | False |
| PneumoniaMNIST | 90%-10% | Supervised | 1.832e-01 | 9.159e-01 | False |
| PneumoniaMNIST | 90%-10% | VICReg | 5.653e-01 | 1 | False |
| **label percentage 5%** | | | | | |
| PneumoniaMNIST | 10%-90% | Supervised | 6.997e-04 | 3.498e-03 | True |
| PneumoniaMNIST | 10%-90% | VICReg | 7.009e-02 | 3.505e-01 | False |
| PneumoniaMNIST | 30%-70% | Supervised | 2.751e-01 | 1 | False |
| PneumoniaMNIST | 30%-70% | VICReg | 3.219e-01 | 1 | False |
| PneumoniaMNIST | 50%-50% | Supervised | 1.051e-04 | 5.254e-04 | True |
| PneumoniaMNIST | 50%-50% | VICReg | 2.055e-02 | 1.027e-01 | False |
| PneumoniaMNIST | 70%-30% | Supervised | 3.792e-02 | 1.896e-01 | False |
| PneumoniaMNIST | 70%-30% | VICReg | 1.220e-01 | 6.098e-01 | False |
| PneumoniaMNIST | 90%-10% | Supervised | 7.284e-04 | 3.642e-03 | True |
| PneumoniaMNIST | 90%-10% | VICReg | 3.761e-01 | 1 | False |

SL, Supervised learning; VICReg, Variance-Invariance-Covariance Regularization.

**Table S4.** For each configuration of label percentage, meta-dataset, class frequency, and learning strategy, 30 experiments have been carried out. We tested if the PR-AUC scores of the 30 experiments under the same configuration were normally distributed using the Shapiro-Wilk test. P-values were adjusted using the Bonferroni correction. If the adjusted p-value < 0.05, we considered the PR-AUC scores not normally distributed.

| **meta-dataset** | **class**  **frequency** | **learning strategy** | **Shapiro-Wilk test p-value** | **Shapiro-Wilk test adjusted p-value** | **deviates from normality** |
| --- | --- | --- | --- | --- | --- |
| **label percentage 100%** | | | | | |
| HEALTHY-LIFESPAN | 10%-90% | Supervised | 5.2071e-04 | 2.6036e-03 | True |
| HEALTHY-LIFESPAN | 10%-90% | VICReg | 8.6185e-01 | 1 | False |
| HEALTHY-LIFESPAN | 30%-70% | Supervised | 2.1905e-01 | 1 | False |
| HEALTHY-LIFESPAN | 30%-70% | VICReg | 7.1533e-01 | 1 | False |
| HEALTHY-LIFESPAN | 50%-50% | Supervised | 2.4174e-02 | 1.2087e-01 | False |
| HEALTHY-LIFESPAN | 50%-50% | VICReg | 6.7486e-01 | 1 | False |
| HEALTHY-LIFESPAN | 70%-30% | Supervised | 1.7970e-05 | 8.9851e-05 | True |
| HEALTHY-LIFESPAN | 70%-30% | VICReg | 4.7327e-01 | 1 | False |
| HEALTHY-LIFESPAN | 90%-10% | Supervised | 5.7664e-01 | 1 | False |
| HEALTHY-LIFESPAN | 90%-10% | VICReg | 4.7836e-01 | 1 | False |
| **label percentage 10%** | | | | | |
| HEALTHY-LIFESPAN | 10%-90% | Supervised | 5.0744e-05 | 2.5372e-04 | True |
| HEALTHY-LIFESPAN | 10%-90% | VICReg | 1.5371e-02 | 7.6854e-02 | True |
| HEALTHY-LIFESPAN | 30%-70% | Supervised | 4.8898e-03 | 2.4449e-02 | True |
| HEALTHY-LIFESPAN | 30%-70% | VICReg | 8.9790e-01 | 1 | False |
| HEALTHY-LIFESPAN | 50%-50% | Supervised | 8.1088e-01 | 1 | False |
| HEALTHY-LIFESPAN | 50%-50% | VICReg | 7.2453e-01 | 1 | False |
| HEALTHY-LIFESPAN | 70%-30% | Supervised | 5.3900e-02 | 2.6950e-01 | False |
| HEALTHY-LIFESPAN | 70%-30% | VICReg | 4.3519e-02 | 2.1759e-01 | False |
| HEALTHY-LIFESPAN | 90%-10% | Supervised | 9.5361e-02 | 4.7681e-01 | False |
| HEALTHY-LIFESPAN | 90%-10% | VICReg | 4.6640e-02 | 2.3320e-01 | False |
| **label percentage 5%** | | | | | |
| HEALTHY-LIFESPAN | 10%-90% | Supervised | 3.9051e-05 | 1.9525e-04 | True |
| HEALTHY-LIFESPAN | 10%-90% | VICReg | 1.5944e-01 | 7.9719e-01 | False |
| HEALTHY-LIFESPAN | 30%-70% | Supervised | 2.4640e-04 | 1.2320e-03 | True |
| HEALTHY-LIFESPAN | 30%-70% | VICReg | 1.2440e-01 | 6.2202e-01 | False |
| HEALTHY-LIFESPAN | 50%-50% | Supervised | 2.0188e-02 | 1.0094e-01 | False |
| HEALTHY-LIFESPAN | 50%-50% | VICReg | 1.6676e-05 | 8.3378e-05 | True |
| HEALTHY-LIFESPAN | 70%-30% | Supervised | 2.7094e-02 | 1.3547e-01 | False |
| HEALTHY-LIFESPAN | 70%-30% | VICReg | 6.4846e-02 | 3.2423e-01 | False |
| HEALTHY-LIFESPAN | 90%-10% | Supervised | 3.4495e-01 | 1 | False |
| HEALTHY-LIFESPAN | 90%-10% | VICReg | 2.3801e-02 | 1.1900e-01 | False |
| **label percentage 100%** | | | | | |
| ADNI | 10%-90% | Supervised | 3.552e-01 | 1 | False |
| ADNI | 10%-90% | VICReg | 7.010e-01 | 1 | False |
| ADNI | 30%-70% | Supervised | 9.853e-01 | 1 | False |
| ADNI | 30%-70% | VICReg | 2.644e-01 | 1 | False |
| ADNI | 50%-50% | Supervised | 7.246e-01 | 1 | False |
| ADNI | 50%-50% | VICReg | 3.318e-01 | 1 | False |
| ADNI | 70%-30% | Supervised | 8.289e-01 | 1 | False |
| ADNI | 70%-30% | VICReg | 3.025e-01 | 1 | False |
| ADNI | 90%-10% | Supervised | 7.763e-01 | 1 | False |
| ADNI | 90%-10% | VICReg | 7.897e-01 | 1 | False |
| **label percentage 10%** | | | | | |
| ADNI | 10%-90% | Supervised | 4.175e-01 | 1 | False |
| ADNI | 10%-90% | VICReg | 6.816e-01 | 1 | False |
| ADNI | 30%-70% | Supervised | 4.021e-02 | 2.010e-01 | False |
| ADNI | 30%-70% | VICReg | 1.224e-01 | 6.124e-01 | False |
| ADNI | 50%-50% | Supervised | 5.664e-01 | 1 | False |
| ADNI | 50%-50% | VICReg | 7.785e-01 | 1 | False |
| ADNI | 70%-30% | Supervised | 9.505e-01 | 1 | False |
| ADNI | 70%-30% | VICReg | 9.465e-01 | 1 | False |
| ADNI | 90%-10% | Supervised | 3.919e-01 | 1 | False |
| ADNI | 90%-10% | VICReg | 9.925e-01 | 1 | False |
| **label percentage 5%** | | | | | |
| ADNI | 10%-90% | Supervised | 3.678e-01 | 1 | False |
| ADNI | 10%-90% | VICReg | 8.349e-01 | 1 | False |
| ADNI | 30%-70% | Supervised | 5.432e-01 | 1 | False |
| ADNI | 30%-70% | VICReg | 7.760e-01 | 1 | False |
| ADNI | 50%-50% | Supervised | 6.096e-01 | 1 | False |
| ADNI | 50%-50% | VICReg | 4.290e-01 | 1 | False |
| ADNI | 70%-30% | Supervised | 8.604e-01 | 1 | False |
| ADNI | 70%-30% | VICReg | 1.853e-01 | 9.267e-01 | False |
| ADNI | 90%-10% | Supervised | 1.737e-01 | 8.684e-01 | False |
| ADNI | 90%-10% | VICReg | 5.541e-01 | 1 | False |
| **label percentage 100%** | | | | | |
| PneumoniaMNIST | 10%-90% | Supervised | 2.909e-01 | 1 | False |
| PneumoniaMNIST | 10%-90% | VICReg | 6.296e-02 | 3.148e-01 | False |
| PneumoniaMNIST | 30%-70% | Supervised | 7.049e-01 | 1 | False |
| PneumoniaMNIST | 30%-70% | VICReg | 8.733e-02 | 4.366e-01 | False |
| PneumoniaMNIST | 50%-50% | Supervised | 9.196e-01 | 1 | False |
| PneumoniaMNIST | 50%-50% | VICReg | 3.584e-01 | 1 | False |
| PneumoniaMNIST | 70%-30% | Supervised | 9.888e-01 | 1 | False |
| PneumoniaMNIST | 70%-30% | VICReg | 2.288e-04 | 1.144e-03 | True |
| PneumoniaMNIST | 90%-10% | Supervised | 2.275e-01 | 1 | False |
| PneumoniaMNIST | 90%-10% | VICReg | 5.845e-01 | 1 | False |
| **label percentage 10%** | | | | | |
| PneumoniaMNIST | 10%-90% | Supervised | 1.073e-05 | 5.366e-05 | True |
| PneumoniaMNIST | 10%-90% | VICReg | 5.633e-02 | 2.817e-01 | False |
| PneumoniaMNIST | 30%-70% | Supervised | 8.000e-01 | 1 | False |
| PneumoniaMNIST | 30%-70% | VICReg | 4.076e-01 | 1 | False |
| PneumoniaMNIST | 50%-50% | Supervised | 6.707e-01 | 1 | False |
| PneumoniaMNIST | 50%-50% | VICReg | 4.976e-01 | 1 | False |
| PneumoniaMNIST | 70%-30% | Supervised | 1.148e-01 | 5.742e-01 | False |
| PneumoniaMNIST | 70%-30% | VICReg | 4.408e-02 | 2.204e-01 | False |
| PneumoniaMNIST | 90%-10% | Supervised | 3.655e-01 | 1 | False |
| PneumoniaMNIST | 90%-10% | VICReg | 2.603e-01 | 1 | False |
| **label percentage 5%** | | | | | |
| PneumoniaMNIST | 10%-90% | Supervised | 2.220e-02 | 1.110e-01 | False |
| PneumoniaMNIST | 10%-90% | VICReg | 1.723e-01 | 8.616e-01 | False |
| PneumoniaMNIST | 30%-70% | Supervised | 2.139e-01 | 1 | False |
| PneumoniaMNIST | 30%-70% | VICReg | 5.834e-02 | 2.917e-01 | False |
| PneumoniaMNIST | 50%-50% | Supervised | 5.568e-04 | 2.784e-03 | True |
| PneumoniaMNIST | 50%-50% | VICReg | 4.926e-02 | 2.463e-01 | False |
| PneumoniaMNIST | 70%-30% | Supervised | 1.452e-02 | 7.262e-02 | False |
| PneumoniaMNIST | 70%-30% | VICReg | 5.598e-02 | 2.799e-01 | False |
| PneumoniaMNIST | 90%-10% | Supervised | 6.288e-04 | 3.144e-03 | True |
| PneumoniaMNIST | 90%-10% | VICReg | 1.366e-01 | 6.828e-01 | False |

PR-AUC, Precision Recall Area Under the Curve; SL, Supervised learning; VICReg, Variance-Invariance-Covariance Regularization.

**Table S5.** Results on the test set across 30 experiments for each class frequency and label percentage configuration of the HEALTHY-LIFESPAN meta-dataset. The mean ROC-AUC and the 2.5^th^ and 97.5^th^ percentiles are reported. The classes frequency column indicates, respectively, the percentage of the negative and positive classes in the training set. The p-value was calculated using a two-tailed paired t-test comparing the metric scores of the two learning strategies when metric scores were normally distributed or a Wilcoxon signed-rank test otherwise. P-values were adjusted using the Bonferroni correction.

| **class frequency** | **SL**  **mean [2.5^th^, 97.5^th^] ROC-AUC** | **VICReg**  **mean [2.5^th^, 97.5^th^]**  **ROC-AUC** | **p-value** | **adjusted**  **p-value** |
| --- | --- | --- | --- | --- |
| **label percentage 100%** | | | | |
| 10%-90% | 0.967 [0.934, 0.980] | 0.916 [0.886, 0.936] | 6.393e-16 | 3.196e-15 |
| 30%-70% | 0.974 [0.963, 0.985] | 0.928 [0.902, 0.945] | 3.984e-20 | 1.992e-19 |
| 50%-50% | 0.977 [0.970, 0.982] | 0.934 [0.913, 0.953] | 5.228e-22 | 2.614e-21 |
| 70%-30% | 0.976 [0.966, 0.982] | 0.936 [0.914, 0.951] | 1.551e-21 | 7.755e-21 |
| 90%-10% | 0.967 [0.949, 0.980] | 0.932 [0.909, 0.954] | 1.491e-16 | 7.455e-16 |
| **label percentage 10%** | | | |  |
| 10%-90% | 0.907 [0.811, 0.952] | 0.842 [0.745, 0.901] | 1.007e-06 | 5.035e-06 |
| 30%-70% | 0.947 [0.909, 0.970] | 0.896 [0.857, 0.929] | 3.777e-15 | 1.889e-14 |
| 50%-50% | 0.948 [0.912, 0.972] | 0.907 [0.875, 0.942] | 1.595e-10 | 7.975e-10 |
| 70%-30% | 0.941 [0.904, 0.966] | 0.908 [0.867, 0.931] | 2.313e-10 | 1.157e-09 |
| 90%-10% | 0.913 [0.875, 0.940] | 0.854 [0.714, 0.933] | 4.898e-06 | 2.449e-05 |
| **label percentage 5%** | | | |  |
| 10%-90% | 0.851 [0.667, 0.926] | 0.790 [0.681, 0.886] | 1.029e-03 | 5.145e-03 |
| 30%-70% | 0.919 [0.856, 0.951] | 0.861 [0.814, 0.902] | 4.450e-10 | 2.225e-09 |
| 50%-50% | 0.924 [0.870, 0.959] | 0.867 [0.743, 0.913] | 1.324e-07 | 6.620e-07 |
| 70%-30% | 0.918 [0.854, 0.953] | 0.861 [0.770, 0.917] | 1.006e-07 | 5.030e-07 |
| 90%-10% | 0.872 [0.793, 0.924] | 0.798 [0.643, 0.886] | 7.400e-06 | 3.700e-05 |

ROC-AUC Receiver Operating Characteristics Area Under the Curve; SL, Supervised learning; VICReg, Variance-Invariance-Covariance Regularization.

**Table S6.** Results on the test set across 30 experiments for each class frequency and label percentage configuration of the ADNI meta-dataset. The mean ROC-AUC and the 2.5^th^ and 97.5^th^ percentiles are reported. The class frequency column indicates the percentage of the negative and positive classes in the training set. The p-value was calculated using a two-tailed paired t-test comparing the metric scores of the two learning strategies when both metric scores were normally distributed or a Wilcoxon signed-rank test otherwise. P-values were adjusted using the Bonferroni correction.

| **class frequency** | **SL**  **mean [2.5^th^, 97.5^th^] ROC-AUC** | **VICReg**  **mean [2.5^th^, 97.5^th^]**  **ROC-AUC** | **p-value** | **adjusted**  **p-value** |
| --- | --- | --- | --- | --- |
| **label percentage 100%** | | | |  |
| 10%-90% | 0.712 [0.636, 0.807] | 0.721 [0.629, 0.790] | 2.576e-01 | 1 |
| 30%-70% | 0.740 [0.657, 0.812] | 0.722 [0.627, 0.788] | 2.110e-02 | 1.055e-01 |
| 50%-50% | 0.746 [0.670, 0.817] | 0.720 [0.630, 0.778] | 5.909e-04 | 2.955e-03 |
| 70%-30% | 0.748 [0.681, 0.813] | 0.719 [0.653, 0.765] | 7.722e-07 | 3.861e-06 |
| 90%-10% | 0.725 [0.661, 0.788] | 0.703 [0.633, 0.783] | 1.425e-02 | 7.125e-02 |
| **label percentage 10%** | | | | |
| 10%-90% | 0.609 [0.460, 0.722] | 0.620 [0.462, 0.753] | 4.346e-01 | 1 |
| 30%-70% | 0.675 [0.548, 0.749] | 0.686 [0.602, 0.751] | 2.345e-01 | 1 |
| 50%-50% | 0.689 [0.553, 0.764] | 0.675 [0.580, 0.747] | 2.488e-01 | 1 |
| 70%-30% | 0.695 [0.574, 0.774] | 0.683 [0.600, 0.743] | 2.021e-01 | 1 |
| 90%-10% | 0.645 [0.514, 0.762] | 0.641 [0.547, 0.724] | 7.858e-01 | 1 |
| **label percentage 5%** | | | |  |
| 10%-90% | 0.582 [0.448, 0.692] | 0.575 [0.384, 0.688] | 6.677e-01 | 1 |
| 30%-70% | 0.629 [0.504, 0.716] | 0.651 [0.541, 0.724] | 6.009e-02 | 3.005e-01 |
| 50%-50% | 0.666 [0.573, 0.753] | 0.656 [0.567, 0.745] | 4.253e-01 | 1 |
| 70%-30% | 0.643 [0.493, 0.732] | 0.646 [0.495, 0.739] | 7.222e-01 | 1 |
| 90%-10% | 0.628 [0.463, 0.733] | 0.602 [0.456, 0.727] | 8.310e-02 | 4.155e-01 |

ROC-AUC Receiver Operating Characteristics Area Under the Curve; SL, Supervised learning; VICReg, Variance-Invariance-Covariance Regularization.

**Table S7.** Results on the test set across 30 experiments for each class frequency and label percentage configuration of the PneumoniaMNIST meta-dataset. The mean ROC-AUC and the 2.5^th^ and 97.5^th^ percentiles are reported. The class frequency column indicates the percentage of the negative and positive classes in the training set. The p-value was calculated using a two-tailed paired t-test comparing the metric scores of the two learning strategies when both metric scores were normally distributed or a Wilcoxon signed-rank test otherwise. P-values were adjusted using the Bonferroni correction.

| **class frequency** | **SL**  **mean [2.5^th^, 97.5^th^] ROC-AUC** | **VICReg**  **mean [2.5^th^, 97.5^th^]**  **ROC-AUC** | **p-value** | **adjusted**  **p-value** |
| --- | --- | --- | --- | --- |
| **label percentage 100%** | | | | |
| 10%-90% | 0.970 [0.956, 0.982] | 0.897 [0.861, 0.930] | 9.427e-19 | 4.714e-18 |
| 30%-70% | 0.977 [0.969, 0.986] | 0.911 [0.884, 0.933] | 1.923e-23 | 9.615e-23 |
| 50%-50% | 0.977 [0.968, 0.987] | 0.914 [0.896, 0.934] | 2.164e-26 | 1.082e-25 |
| 70%-30% | 0.975 [0.962, 0.987] | 0.913 [0.886, 0.932] | 1.446e-24 | 7.230e-24 |
| 90%-10% | 0.970 [0.958, 0.982] | 0.909 [0.887, 0.927] | 1.130e-22 | 5.650e-22 |
| **label percentage 10%** | | | | |
| 10%-90% | 0.940 [0.898, 0.963] | 0.843 [0.701, 0.913] | 2.805e-12 | 1.403e-11 |
| 30%-70% | 0.945 [0.929, 0.967] | 0.879 [0.818, 0.931] | 9.921e-13 | 4.960e-12 |
| 50%-50% | 0.947 [0.925, 0.968] | 0.886 [0.854, 0.914] | 7.053e-21 | 3.527e-20 |
| 70%-30% | 0.946 [0.920, 0.966] | 0.887 [0.842, 0.923] | 5.072e-16 | 2.536e-15 |
| 90%-10% | 0.939 [0.913, 0.960] | 0.870 [0.820, 0.911] | 7.258e-15 | 3.629e-14 |
| **label percentage 5%** | | | |  |
| 10%-90% | 0.925 [0.858, 0.962] | 0.801 [0.673, 0.881] | 1.740e-13 | 8.700e-13 |
| 30%-70% | 0.941 [0.914, 0.958] | 0.857 [0.777, 0.914] | 2.120e-13 | 1.060e-12 |
| 50%-50% | 0.940 [0.891, 0.962] | 0.873 [0.802, 0.911] | 1.223e-14 | 6.115e-14 |
| 70%-30% | 0.934 [0.908, 0.956] | 0.858 [0.784, 0.903] | 2.177e-13 | 1.089e-12 |
| 90%-10% | 0.926 [0.862, 0.954] | 0.837 [0.769, 0.883] | 1.890e-14 | 9.450e-14 |

ROC-AUC Receiver Operating Characteristics Area Under the Curve; SL, Supervised learning; VICReg, Variance-Invariance-Covariance Regularization.

**Table S8.** Results on the test set across 5 experiments for each class frequency and label percentage configuration of the OCTMNIST meta-dataset. The mean ROC-AUC is reported. The class frequency column indicates, respectively, the percentage of the negative and positive classes in the training set.

| **class frequency** | **SL**  **mean ROC-AUC** | **VICReg**  **mean ROC-AUC** | **MAE**  **mean ROC-AUC** | |  |  |
| --- | --- | --- | --- | --- | --- | --- |
| **label percentage 100%** | | | | |  |  |
| 10%-90% | 0.998 | 0.975 | 0.945 | |  |  |
| 30%-70% | 0.999 | 0.987 | 0.972 | |  |  |
| 50%-50% | 0.999 | 0.989 | 0.980 | |  |  |
| 70%-30% | 0.999 | 0.989 | 0.986 | |  |  |
| 90%-10% | 0.999 | 0.976 | 0.986 | |  |  |
| **label percentage 10%** | | | | |  |  |
| 10%-90% | 0.991 | 0.930 | 0.863 | |  |  |
| 30%-70% | 0.997 | 0.971 | 0.941 | |  |  |
| 50%-50% | 0.998 | 0.979 | 0.953 | |  |  |
| 70%-30% | 0.997 | 0.984 | 0.970 | |  |  |
| 90%-10% | 0.995 | 0.970 | 0.967 | |  |  |
| **label percentage 5%** | | | |  |  |  |
| 10%-90% | | 0.878 | 0.816 | 0.845 | |  |
| 30%-70% | | 0.994 | 0.925 | 0.926 | |  |
| 50%-50% | | 0.995 | 0.962 | 0.936 | |  |
| 70%-30% | | 0.995 | 0.979 | 0.960 | |  |
| 90%-10% | | 0.973 | 0.964 | 0.953 | |  |
| **label percentage 1%** | | | |  |  |  |
| 10%-90% | 0.449 | 0.592 | 0.661 | |  |  |
| 30%-70% | 0.460 | 0.678 | 0.883 | |  |  |
| 50%-50% | 0.493 | 0.751 | 0.907 | |  |  |
| 70%-30% | 0.525 | 0.822 | 0.938 | |  |  |
| 90%-10% | 0.554 | 0.877 | 0.904 | |  |  |

MAE, Masked Autoencoder; ROC-AUC, Precision Recall Area Under the Curve; SL, Supervised learning; VICReg, Variance-Invariance-Covariance Regularization.

**Table S9.** Results on the test set across 30 experiments for each class frequency and label percentage configuration of the HEALTHY-LIFESPAN meta-dataset. The mean PR-AUC and the 2.5^th^ and 97.5^th^ percentiles are reported. The class frequency column indicates, respectively, the percentage of the negative and positive classes in the training set. The p-value was calculated using a two-tailed paired t-test comparing the metric scores of the two learning strategies when metric scores were normally distributed or a Wilcoxon signed-rank test otherwise. P-values were adjusted using the Bonferroni correction.

| **class frequency** | **SL**  **mean [2.5^th^, 97.5^th^] PR-AUC** | **VICReg**  **mean [2.5^th^, 97.5^th^]**  **PR-AUC** | **p-value** | **adjusted**  **p-value** |
| --- | --- | --- | --- | --- |
| **label percentage 100%** | | | | |
| 10%-90% | 0.957 [0.902, 0.978] | 0.912 [0.883, 0.940] | 2.353e-06 | 1.177e-05 |
| 30%-70% | 0.969 [0.948, 0.985] | 0.924 [0.899, 0.945] | 5.724e-18 | 2.862e-17 |
| 50%-50% | 0.975 [0.962, 0.983] | 0.930 [0.904, 0.953] | 1.148e-20 | 5.738e-20 |
| 70%-30% | 0.974 [0.953, 0.982] | 0.931 [0.909, 0.951] | 1.734e-06 | 8.672e-06 |
| 90%-10% | 0.969 [0.953, 0.979] | 0.925 [0.895, 0.951] | 9.202e-16 | 4.601e-15 |
| **label percentage 10%** | | | |  |
| 10%-90% | 0.886 [0.722, 0.950] | 0.826 [0.692, 0.918] | 2.255e-03 | 1.128e-02 |
| 30%-70% | 0.937 [0.899, 0.966] | 0.891 [0.855, 0.928] | 1.734e-06 | 8.672e-06 |
| 50%-50% | 0.946 [0.914, 0.974] | 0.904 [0.870, 0.938] | 2.824e-10 | 1.412e-09 |
| 70%-30% | 0.941 [0.905, 0.966] | 0.907 [0.876, 0.927] | 1.572e-12 | 7.858e-12 |
| 90%-10% | 0.919 [0.889, 0.949] | 0.863 [0.745, 0.923] | 7.034e-07 | 3.517e-06 |
| **label percentage 5%** | | | |  |
| 10%-90% | 0.826 [0.574, 0.929] | 0.764 [0.600, 0.884] | 7.271e-03 | 3.636e-02 |
| 30%-70% | 0.917 [0.826, 0.955] | 0.853 [0.806, 0.907] | 2.879e-06 | 1.439e-05 |
| 50%-50% | 0.925 [0.865, 0.956] | 0.863 [0.722, 0.908] | 4.729e-06 | 2.365e-05 |
| 70%-30% | 0.919 [0.868, 0.951] | 0.861 [0.778, 0.914] | 1.961e-08 | 9.805e-08 |
| 90%-10% | 0.885 [0.828, 0.932] | 0.812 [0.685, 0.896] | 1.423e-06 | 7.116e-06 |

PR-AUC, Precision Recall Area Under the Curve; SL, Supervised learning; VICReg, Variance-Invariance-Covariance Regularization.

**Table S10.** Results on the test set across 30 experiments for each class frequency and label percentage configuration of the ADNI meta-dataset. The mean PR-AUC and the 2.5^th^ and 97.5^th^ percentiles are reported. The class frequency column indicates the percentage of the negative and positive classes in the training set. The p-value was calculated using a two-tailed paired t-test comparing the metric scores of the two learning strategies when both metric scores were normally distributed or a Wilcoxon signed-rank test otherwise. P-values were adjusted using the Bonferroni correction.

| **class frequency** | **SL**  **mean [2.5^th^, 97.5^th^] PR-AUC** | **VICReg**  **mean [2.5^th^, 97.5^th^]**  **PR-AUC** | **p-value** | **adjusted**  **p-value** |
| --- | --- | --- | --- | --- |
| **label percentage 100%** | | | |  |
| 10%-90% | 0.7 [0.623, 0.811] | 0.713 [0.613, 0.804] | 1.406e-01 | 7.032e-01 |
| 30%-70% | 0.73 [0.631, 0.839] | 0.711 [0.620, 0.787] | 5.490e-02 | 2.745e-01 |
| 50%-50% | 0.739 [0.671, 0.817] | 0.709 [0.620, 0.781] | 7.440e-04 | 3.720e-03 |
| 70%-30% | 0.735 [0.668, 0.805] | 0.711 [0.656, 0.774] | 8.304e-04 | 4.152e-03 |
| 90%-10% | 0.721 [0.642, 0.790] | 0.695 [0.607, 0.789] | 8.737e-03 | 4.368e-02 |
| **label percentage 10%** | | | | |
| 10%-90% | 0.587 [0.474, 0.720] | 0.608 [0.465, 0.725] | 1.753e-01 | 8.764e-01 |
| 30%-70% | 0.652 [0.533, 0.723] | 0.671 [0.581, 0.751] | 4.411e-02 | 2.205e-01 |
| 50%-50% | 0.675 [0.549, 0.766] | 0.665 [0.572, 0.758] | 4.315e-01 | 1 |
| 70%-30% | 0.683 [0.582, 0.774] | 0.674 [0.593, 0.751] | 3.672e-01 | 1 |
| 90%-10% | 0.634 [0.508, 0.770] | 0.632 [0.531, 0.727] | 8.498e-01 | 1 |
| **label percentage 5%** | | | |  |
| 10%-90% | 0.563 [0.462, 0.697] | 0.565 [0.425, 0.677] | 9.432e-01 | 1 |
| 30%-70% | 0.609 [0.496, 0.724] | 0.631 [0.526, 0.738] | 2.784e-02 | 1.392e-01 |
| 50%-50% | 0.652 [0.564, 0.750] | 0.652 [0.550, 0.719] | 9.966e-01 | 1 |
| 70%-30% | 0.626 [0.498, 0.734] | 0.643 [0.505, 0.744] | 1.349e-01 | 6.747e-01 |
| 90%-10% | 0.623 [0.472, 0.733] | 0.605 [0.468, 0.718] | 1.511e-01 | 7.556e-01 |

PR-AUC, Precision Recall Area Under the Curve; SL, Supervised learning; VICReg, Variance-Invariance-Covariance Regularization.

**Table S11.** Results on the test set across 30 experiments for each class frequency and label percentage configuration of the PneumoniaMNIST meta-dataset. The mean PR-AUC and the 2.5^th^ and 97.5^th^ percentiles are reported. The class frequency column indicates the percentage of the negative and positive classes in the training set. The p-value was calculated using a two-tailed paired t-test comparing the metric scores of the two learning strategies when both metric scores were normally distributed or a Wilcoxon signed-rank test otherwise. P-values were adjusted using the Bonferroni correction.

| **class frequency** | **SL**  **mean [2.5^th^, 97.5^th^] PR-AUC** | **VICReg**  **mean [2.5^th^, 97.5^th^]**  **PR-AUC** | **p-value** | **adjusted**  **p-value** |
| --- | --- | --- | --- | --- |
| **label percentage 100%** | | | | |
| 10%-90% | 0.969 [0.954, 0.981] | 0.871 [0.804, 0.924] | 8.677e-15 | 4.339e-14 |
| 30%-70% | 0.975 [0.966, 0.987] | 0.897 [0.845, 0.940] | 5.159e-18 | 2.579e-17 |
| 50%-50% | 0.976 [0.965, 0.987] | 0.903 [0.862, 0.930] | 4.549e-21 | 2.274e-20 |
| 70%-30% | 0.973 [0.962, 0.987] | 0.907 [0.863, 0.931] | 1.734e-06 | 8.672e-06 |
| 90%-10% | 0.968 [0.953, 0.981] | 0.904 [0.874, 0.930] | 1.992e-19 | 9.959e-19 |
| **label percentage 10%** | | | | |
| 10%-90% | 0.941 [0.891, 0.967] | 0.797 [0.614, 0.905] | 1.734e-06 | 8.672e-06 |
| 30%-70% | 0.944 [0.920, 0.966] | 0.852 [0.736, 0.933] | 2.653e-10 | 1.326e-09 |
| 50%-50% | 0.945 [0.916, 0.969] | 0.862 [0.792, 0.923] | 1.489e-14 | 7.447e-14 |
| 70%-30% | 0.944 [0.914, 0.965] | 0.876 [0.792, 0.925] | 8.936e-13 | 4.468e-12 |
| 90%-10% | 0.942 [0.919, 0.962] | 0.858 [0.783, 0.921] | 1.122e-12 | 5.612e-12 |
| **label percentage 5%** | | | |  |
| 10%-90% | 0.924 [0.868, 0.964] | 0.745 [0.579, 0.862] | 1.057e-13 | 5.285e-13 |
| 30%-70% | 0.942 [0.918, 0.962] | 0.824 [0.678, 0.907] | 2.357e-11 | 1.179e-10 |
| 50%-50% | 0.941 [0.895, 0.961] | 0.848 [0.750, 0.909] | 1.734e-06 | 8.672e-06 |
| 70%-30% | 0.932 [0.882, 0.960] | 0.837 [0.713, 0.903] | 5.987e-13 | 2.994e-12 |
| 90%-10% | 0.929 [0.867, 0.958] | 0.813 [0.724, 0.884] | 1.734e-06 | 8.672e-06 |

PR-AUC, Precision Recall Area Under the Curve; SL, Supervised learning; VICReg, Variance-Invariance-Covariance Regularization.

**Table S12.** Results on the test set across 5 experiments for each class frequency and label percentage configuration of the OCTMNIST meta-dataset. The mean PR-AUC is reported. The class frequency column indicates, respectively, the percentage of the negative and positive classes in the training set.

| **class frequency** | **SL**  **mean PR-AUC** | **VICReg**  **mean PR-AUC** | **MAE**  **mean PR-AUC** | |  |  |
| --- | --- | --- | --- | --- | --- | --- |
| **label percentage 100%** | | | | |  |  |
| 10%-90% | 0.996 | 0.975 | 0.947 | |  |  |
| 30%-70% | 0.999 | 0.986 | 0.972 | |  |  |
| 50%-50% | 0.999 | 0.989 | 0.980 | |  |  |
| 70%-30% | 0.999 | 0.989 | 0.987 | |  |  |
| 90%-10% | 0.999 | 0.977 | 0.987 | |  |  |
| **label percentage 10%** | | | | |  |  |
| 10%-90% | 0.984 | 0.923 | 0.862 | |  |  |
| 30%-70% | 0.997 | 0.970 | 0.936 | |  |  |
| 50%-50% | 0.998 | 0.980 | 0.951 | |  |  |
| 70%-30% | 0.997 | 0.984 | 0.970 | |  |  |
| 90%-10% | 0.996 | 0.972 | 0.968 | |  |  |
| **label percentage 5%** | | | |  |  |  |
| 10%-90% | | 0.886 | 0.804 | 0.840 | |  |
| 30%-70% | | 0.994 | 0.924 | 0.914 | |  |
| 50%-50% | | 0.995 | 0.962 | 0.933 | |  |
| 70%-30% | | 0.995 | 0.980 | 0.957 | |  |
| 90%-10% | | 0.975 | 0.966 | 0.952 | |  |
| **label percentage 1%** | | | |  |  |  |
| 10%-90% | 0.463 | 0.610 | 0.662 | |  |  |
| 30%-70% | 0.469 | 0.679 | 0.868 | |  |  |
| 50%-50% | 0.495 | 0.741 | 0.900 | |  |  |
| 70%-30% | 0.519 | 0.808 | 0.933 | |  |  |
| 90%-10% | 0.545 | 0.878 | 0.894 | |  |  |

MAE, Masked Autoencoder; PR-AUC, Precision Recall Area Under the Curve; SL, Supervised learning; VICReg, Variance-Invariance-Covariance Regularization.

**Table S13.** Results on the test set across 30 experiments for each class frequency and label percentage configuration of the HEALTHY-LIFESPAN meta-dataset. The class frequency column indicates, respectively, the percentage of the negative and positive classes in the training set. It is reported the mean difference between each class imbalance configuration and the respective balanced one (50%-50%). The p-value was calculated using a two-tailed paired t-test comparing the ROC-AUC metric scores of the two learning strategies when both metric scores were normally distributed or a Wilcoxon signed-rank test otherwise. The p-values were adjusted using Bonferroni correction.

| **class frequency** | **SL**  **mean**  **ROC-AUC difference** | **SL**  **p-value** | **SL**  **adjusted**  **p-value** | **VICReg**  **mean**  **ROC-AUC difference** | | **VICReg**  **p-value** | **VICReg**  **adjusted**  **p-value** |
| --- | --- | --- | --- | --- | --- | --- | --- |
| **label percentage 100%** | | | | |  |  |  |
| 10%-90% | -0.00969 | 4.286e-06 | 3.429e-05 | -0.01858 | | 1.247e-09 | 9.979e-09 |
| 30%-70% | -0.00273 | 6.678e-03 | 5.342e-02 | -0.00629 | | 3.668e-04 | 2.934e-03 |
| 70%-30% | -0.00059 | 5.136e-01 | 1 | 0.00158 | | 3.217e-01 | 1 |
| 90%-10% | -0.00947 | 1.084e-07 | 8.675e-07 | -0.00238 | | 2.002e-01 | 1 |
| **label percentage 10%** | | | | |  |  |  |
| 10%-90% | -0.04098 | 1.360e-05 | 1.088e-04 | -0.06480 | | 2.809e-07 | 2.247e-06 |
| 30%-70% | -0.00143 | 7.389e-01 | 1 | -0.01123 | | 4.215e-02 | 3.372e-01 |
| 70%-30% | -0.00698 | 1.270e-01 | 1 | 0.00088 | | 8.112e-01 | 1 |
| 90%-10% | -0.03517 | 1.054e-07 | 8.428e-07 | -0.05285 | | 2.128e-05 | 1.702e-04 |
| **label percentage 5%** | | | | |  |  |  |
| 10%-90% | -0.07334 | 7.691e-06 | 6.153e-05 | -0.07727 | | 8.466e-06 | 6.773e-05 |
| 30%-70% | -0.00502 | 5.857e-01 | 1 | -0.00628 | | 4.528e-01 | 1 |
| 70%-30% | -0.00672 | 3.418e-01 | 1 | -0.00625 | | 4.284e-01 | 1 |
| 90%-10% | -0.05234 | 6.395e-07 | 5.116e-06 | -0.06920 | | 6.156e-04 | 4.925e-03 |

ROC-AUC Receiver Operating Characteristics Area Under the Curve; SL, Supervised learning; VICReg, Variance-Invariance-Covariance Regularization.

**Table S14.** Results on the test set across 30 experiments for each class frequency and label percentage configuration of the ADNI meta-dataset. The classes frequency column indicates, respectively, the percentage of the negative and positive classes in the training set. It is reported the mean difference between each class imbalance configuration and the respective balanced one (50%-50%). The p-value was calculated using a two-tailed paired t-test comparing the ROC-AUC metric scores when both metric scores were normally distributed or a Wilcoxon signed-rank test otherwise. The p-values were adjusted using Bonferroni correction.

| **class frequency** | **SL**  **mean**  **ROC-AUC difference** | **SL**  **p-value** | **SL**  **adjusted**  **p-value** | **VICReg**  **mean**  **ROC-AUC difference** | | **VICReg**  **p-value** | **VICReg**  **adjusted**  **p-value** |
| --- | --- | --- | --- | --- | --- | --- | --- |
| **label percentage 100%** | | | | |  |  |  |
| 10%-90% | -0.03463 | 8.388e-06 | 6.711e-05 | 0.00040 | | 9.312e-01 | 1 |
| 30%-70% | -0.00694 | 2.509e-01 | 1 | 0.00167 | | 6.777e-01 | 1 |
| 70%-30% | 0.00192 | 6.596e-01 | 1 | -0.00121 | | 7.085e-01 | 1 |
| 90%-10% | -0.02102 | 7.373e-03 | 5.899e-02 | -0.01684 | | 4.855e-03 | 3.884e-02 |
| **label percentage 10%** | | | | |  |  |  |
| 10%-90% | -0.08043 | 2.412e-04 | 1.929e-03 | -0.05535 | | 1.525e-03 | 1.220e-02 |
| 30%-70% | -0.01372 | 3.600e-01 | 1 | 0.01066 | | 2.520e-01 | 1 |
| 70%-30% | 0.00557 | 8.612e-01 | 1 | 0.00778 | | 3.756e-01 | 1 |
| 90%-10% | -0.04455 | 1.044e-02 | 8.356e-02 | -0.03379 | | 2.795e-03 | 2.236e-02 |
| **label percentage 5%** | | | | |  |  |  |
| 10%-90% | -0.08461 | 3.247e-05 | 2.598e-04 | -0.08112 | | 1.208e-04 | 9.668e-04 |
| 30%-70% | -0.03727 | 3.555e-02 | 2.844e-01 | -0.00584 | | 6.640e-01 | 1 |
| 70%-30% | -0.02360 | 1.308e-01 | 1 | -0.01010 | | 4.681e-01 | 1 |
| 90%-10% | -0.03789 | 1.498e-02 | 1.198e-01 | -0.05406 | | 6.700e-03 | 5.360e-02 |

ROC-AUC Receiver Operating Characteristics Area Under the Curve; SL, Supervised learning; VICReg, Variance-Invariance-Covariance Regularization.

**Table S15.** Results on the test set across 30 experiments for each class frequency and label percentage configuration of the PneumoniaMNIST meta-dataset. The class frequency column indicates, respectively, the percentage of the negative and positive classes in the training set. It is reported the mean difference between each class imbalance configuration and the respective balanced one (50%-50%). The p-value was calculated using a two-tailed paired t-test when the ROC-AUC metric scores were normally distributed or a Wilcoxon signed-rank test otherwise. The p-values were adjusted using Bonferroni correction.

| **class frequency** | **SL**  **mean**  **ROC-AUC difference** | **SL**  **p-value** | **SL**  **adjusted**  **p-value** | **VICReg**  **mean**  **ROC-AUC difference** | | **VICReg**  **p-value** | **VICReg**  **adjusted**  **p-value** |
| --- | --- | --- | --- | --- | --- | --- | --- |
| **label percentage 100%** | | | | |  |  |  |
| 10%-90% | -0.00748 | 2.609e-07 | 2.087e-06 | -0.01712 | | 5.413e-07 | 4.330e-06 |
| 30%-70% | -0.00077 | 2.810e-01 | 1 | -0.00306 | | 4.015e-02 | 3.212e-01 |
| 70%-30% | -0.00250 | 1.438e-03 | 1.150e-02 | -0.00112 | | 5.204e-01 | 1 |
| 90%-10% | -0.00737 | 1.864e-06 | 1.491e-05 | -0.00509 | | 2.085e-02 | 1.668e-01 |
| **label percentage 10%** | | | | |  |  |  |
| 10%-90% | -0.00655 | 1.156e-01 | 9.249e-01 | -0.04291 | | 2.225e-04 | 1.780e-03 |
| 30%-70% | -0.00144 | 5.182e-01 | 1 | -0.00634 | | 3.323e-01 | 1 |
| 70%-30% | -0.00091 | 7.292e-01 | 1 | 0.00142 | | 7.675e-01 | 1 |
| 90%-10% | -0.00745 | 2.554e-02 | 2.043e-01 | -0.01571 | | 1.436e-02 | 1.149e-01 |
| **label percentage 5%** | | | | |  |  |  |
| 10%-90% | -0.01488 | 6.035e-03 | 4.828e-02 | -0.07196 | | 2.576e-06 | 2.061e-05 |
| 30%-70% | 0.00099 | 7.813e-01 | 1 | -0.01597 | | 8.071e-02 | 6.457e-01 |
| 70%-30% | -0.00530 | 1.414e-01 | 1 | -0.01446 | | 6.205e-02 | 4.964e-01 |
| 90%-10% | -0.01328 | 1.657e-02 | 1.325e-01 | -0.03556 | | 1.493e-04 | 1.194e-03 |

ROC-AUC Receiver Operating Characteristics Area Under the Curve; SL, Supervised learning; VICReg, Variance-Invariance-Covariance Regularization.

**Table S16.** Results on the test set across 5 experiments for each class frequency and label percentage configuration of the OCTMNIST meta-dataset. The class frequency column indicates, respectively, the percentage of the negative and positive classes in the training set. It is reported the mean ROC-AUC difference between each class imbalance configuration and the respective balanced one (50%-50%).

| **class frequency** | **SL**  **mean ROC-AUC**  **difference** | **VICReg**  **mean ROC-AUC**  **ROC-AUC** | **MAE**  **mean ROC-AUC**  **ROC-AUC** | |  |  |
| --- | --- | --- | --- | --- | --- | --- |
| **label percentage 100%** | | | | |  |  |
| 10%-90% | -1.741e-03 | -1.402e-02 | -3.459e-02 | |  |  |
| 30%-70% | -1.322e-04 | -2.126e-03 | -8.037e-03 | |  |  |
| 70%-30% | -1.749e-05 | 4.615e-04 | 6.617e-03 | |  |  |
| 90%-10% | -2.334e-04 | -1.316e-02 | 6.608e-03 | |  |  |
| **label percentage 10%** | | | | |  |  |
| 10%-90% | -6.610e-03 | -4.936e-02 | -8.969e-02 | |  |  |
| 30%-70% | -1.246e-03 | -7.904e-03 | -1.172e-02 | |  |  |
| 70%-30% | -5.479e-04 | 4.666e-03 | 1.753e-02 | |  |  |
| 90%-10% | -3.163e-03 | -9.470e-03 | 1.452e-02 | |  |  |
| **label percentage 5%** | | | |  |  |  |
| 10%-90% | | -1.178e-01 | -1.457e-01 | -9.111e-02 | |  |
| 30%-70% | | -1.268e-03 | -3.642e-02 | -9.831e-03 | |  |
| 70%-30% | | -3.813e-04 | 1.687e-02 | 2.415e-02 | |  |
| 90%-10% | | -2.282e-02 | 2.314e-03 | 1.694e-02 | |  |
| **label percentage 1%** | | | |  |  |  |
| 10%-90% | -4.400e-02 | -1.585e-01 | -2.454e-01 | |  |  |
| 30%-70% | -3.264e-02 | -7.251e-02 | -2.351e-02 | |  |  |
| 70%-30% | 3.175e-02 | 7.095e-02 | 3.140e-02 | |  |  |
| 90%-10% | 6.086e-02 | 1.269e-01 | -3.011e-03 | |  |  |

MAE, Masked Autoencoder; ROC-AUC, Precision Recall Area Under the Curve; SL, Supervised learning; VICReg, Variance-Invariance-Covariance Regularization.

**Table S17.** Results on the test set across 30 experiments for each class frequency and label percentage configuration of the HEALTHY-LIFESPAN meta-dataset. The class frequency column indicates, respectively, the percentage of the negative and positive classes in the training set. It is reported the mean difference between each class imbalance configuration and the respective balanced one (50%-50%). The p-value was calculated using a two-tailed paired t-test comparing the PR-AUC metric scores when both metric scores were normally distributed or a Wilcoxon signed-rank test otherwise. The p-values were adjusted using Bonferroni correction.

| **class frequency** | **SL**  **mean**  **PR-AUC difference** | **SL**  **p-value** | **SL**  **adjusted**  **p-value** | **VICReg**  **mean**  **PR-AUC difference** | | **VICReg**  **p-value** | **VICReg**  **adjusted**  **p-value** |
| --- | --- | --- | --- | --- | --- | --- | --- |
| **label percentage 100%** | | | | |  |  |  |
| 10%-90% | -0.01784 | 2.603e-06 | 2.083e-05 | -0.01850 | | 7.552e-06 | 6.042e-05 |
| 30%-70% | -0.00544 | 2.040e-03 | 1.632e-02 | -0.00606 | | 1.605e-02 | 1.284e-01 |
| 70%-30% | -0.00029 | 8.612e-01 | 1 | 0.00116 | | 6.035e-01 | 1 |
| 90%-10% | -0.00586 | 4.152e-04 | 3.322e-03 | -0.00479 | | 9.276e-02 | 7.421e-01 |
| **label percentage 10%** | | | | |  |  |  |
| 10%-90% | -0.05995 | 6.984e-06 | 5.587e-05 | -0.07781 | | 1.816e-06 | 1.453e-05 |
| 30%-70% | -0.00814 | 5.446e-02 | 4.357e-01 | -0.01295 | | 2.482e-02 | 1.986e-01 |
| 70%-30% | -0.00412 | 2.957e-01 | 1 | 0.00244 | | 5.377e-01 | 1 |
| 90%-10% | -0.02677 | 4.067e-06 | 3.253e-05 | -0.04153 | | 1.380e-04 | 1.104e-03 |
| **label percentage 5%** | | | | |  |  |  |
| 10%-90% | -0.09926 | 5.216e-06 | 4.173e-05 | -0.09884 | | 7.691e-06 | 6.153e-05 |
| 30%-70% | -0.00823 | 3.709e-01 | 1 | -0.00925 | | 3.185e-01 | 1 |
| 70%-30% | -0.00565 | 3.495e-01 | 1 | -0.00202 | | 3.493e-01 | 1 |
| 90%-10% | -0.03992 | 1.800e-06 | 1.440e-05 | -0.05055 | | 5.320e-03 | 4.256e-02 |

PR-AUC, Precision Recall Area Under the Curve; SL, Supervised learning; VICReg, Variance-Invariance-Covariance Regularization.

**Table S18.** Results on the test set across 30 experiments for each class frequency and label percentage configuration of the ADNI meta-dataset. The class frequency column indicates, respectively, the percentage of the negative and positive classes in the training set. It is reported the mean difference between each class imbalance configuration and the respective balanced one (50%-50%). The p-value was calculated using a two-tailed paired t-test comparing the PR-AUC metric scores when both metric scores were normally distributed or a Wilcoxon signed-rank test otherwise. The p-values were adjusted using Bonferroni correction.

| **class frequency** | **SL**  **mean**  **PR-AUC difference** | **SL**  **p-value** | **SL**  **adjusted**  **p-value** | **VICReg**  **mean**  **PR-AUC difference** | | **VICReg**  **p-value** | **VICReg**  **adjusted**  **p-value** |
| --- | --- | --- | --- | --- | --- | --- | --- |
| **label percentage 100%** | | | | |  |  |  |
| 10%-90% | -0.03869 | 8.715e-06 | 6.972e-05 | 0.00395 | | 5.340e-01 | 1 |
| 30%-70% | -0.00910 | 2.059e-01 | 1 | 0.00164 | | 7.584e-01 | 1 |
| 70%-30% | -0.00385 | 4.341e-01 | 1 | 0.00142 | | 8.269e-01 | 1 |
| 90%-10% | -0.01770 | 2.661e-02 | 2.129e-01 | -0.01385 | | 5.823e-02 | 4.659e-01 |
| **label percentage 10%** | | | | |  |  |  |
| 10%-90% | -0.08817 | 9.608e-06 | 7.686e-05 | -0.05766 | | 8.323e-04 | 6.659e-03 |
| 30%-70% | -0.02339 | 1.472e-01 | 1 | 0.00623 | | 4.988e-01 | 1 |
| 70%-30% | 0.00797 | 5.833e-01 | 1 | 0.00830 | | 3.869e-01 | 1 |
| 90%-10% | -0.04135 | 3.250e-02 | 2.600e-01 | -0.03358 | | 1.072e-02 | 8.572e-02 |
| **label percentage 5%** | | | | |  |  |  |
| 10%-90% | -0.08868 | 1.037e-05 | 8.298e-05 | -0.08758 | | 8.320e-07 | 6.656e-06 |
| 30%-70% | -0.04356 | 2.079e-02 | 1.663e-01 | -0.02083 | | 1.903e-01 | 1 |
| 70%-30% | -0.02579 | 1.047e-01 | 8.378e-01 | -0.00874 | | 5.990e-01 | 1 |
| 90%-10% | -0.02914 | 5.186e-02 | 4.149e-01 | -0.04702 | | 1.065e-02 | 8.524e-02 |

PR-AUC, Precision Recall Area Under the Curve; SL, Supervised learning; VICReg, Variance-Invariance-Covariance Regularization.

**Table S19.** Results on the test set across 30 experiments for each class frequency and label percentage configuration of the PneumoniaMNIST meta-dataset. The class frequency column indicates, respectively, the percentage of the negative and positive classes in the training set. It is reported the mean difference between each class imbalance configuration and the respective balanced one (50%-50%). The p-value was calculated using a two-tailed paired t-test comparing the PR-AUC metric scores when both metric scores were normally distributed or a Wilcoxon signed-rank test otherwise. The p-values were adjusted using Bonferroni correction.

| **class frequency** | **SL**  **mean**  **PR-AUC difference** | **SL**  **p-value** | **SL**  **adjusted**  **p-value** | **VICReg**  **mean**  **PR-AUC difference** | | **VICReg**  **p-value** | **VICReg**  **adjusted**  **p-value** |
| --- | --- | --- | --- | --- | --- | --- | --- |
| **label percentage 100%** | | | | |  |  |  |
| 10%-90% | -0.00740 | 1.126e-05 | 9.008e-05 | -0.03213 | | 1.861e-05 | 1.489e-04 |
| 30%-70% | -0.00112 | 1.852e-01 | 1 | -0.00610 | | 5.436e-02 | 4.349e-01 |
| 70%-30% | -0.00282 | 5.644e-03 | 4.515e-02 | 0.00366 | | 2.802e-01 | 1 |
| 90%-10% | -0.00806 | 3.116e-05 | 2.493e-04 | 0.00106 | | 7.738e-01 | 1 |
| **label percentage 10%** | | | | |  |  |  |
| 10%-90% | -0.00380 | 5.170e-01 | 1 | -0.06499 | | 1.963e-04 | 1.570e-03 |
| 30%-70% | -0.00103 | 7.317e-01 | 1 | -0.00974 | | 4.253e-01 | 1 |
| 70%-30% | -0.00141 | 6.229e-01 | 1 | 0.01425 | | 1.441e-01 | 1 |
| 90%-10% | -0.00326 | 3.012e-01 | 1 | -0.00364 | | 7.416e-01 | 1 |
| **label percentage 5%** | | | | |  |  |  |
| 10%-90% | -0.01645 | 3.379e-03 | 2.703e-02 | -0.10323 | | 8.069e-06 | 6.455e-05 |
| 30%-70% | 0.00125 | 9.918e-01 | 1 | -0.02424 | | 1.152e-01 | 9.219e-01 |
| 70%-30% | -0.00807 | 1.470e-01 | 1 | -0.01066 | | 3.920e-01 | 1 |
| 90%-10% | -0.01180 | 4.950e-02 | 3.960e-01 | -0.03534 | | 1.134e-02 | 9.070e-02 |

PR-AUC, Precision Recall Area Under the Curve; SL, Supervised learning; VICReg, Variance-Invariance-Covariance Regularization.

**Table S20.** Results on the test set across 5 experiments for each class frequency and label percentage configuration of the OCTMNIST meta-dataset. The class frequency column indicates, respectively, the percentage of the negative and positive classes in the training set. It is reported the mean PR-AUC difference between each class imbalance configuration and the respective balanced one (50%-50%).

| **class frequency** | **SL**  **mean PR-AUC**  **difference** | **VICReg**  **mean PR-AUC**  **difference** | **MAE**  **mean PR-AUC**  **difference** | |  |  |
| --- | --- | --- | --- | --- | --- | --- |
| **label percentage 100%** | | | | |  |  |
| 10%-90% | -3.239e-03 | -1.415e-02 | -3.335e-02 | |  |  |
| 30%-70% | -1.958e-04 | -2.861e-03 | -8.223e-03 | |  |  |
| 70%-30% | -7.978e-06 | 1.504e-04 | 6.271e-03 | |  |  |
| 90%-10% | -8.105e-05 | -1.217e-02 | 6.533e-03 | |  |  |
| **label percentage 10%** | | | | |  |  |
| 10%-90% | -1.387e-02 | -5.662e-02 | -8.829e-02 | |  |  |
| 30%-70% | -1.212e-03 | -1.017e-02 | -1.488e-02 | |  |  |
| 70%-30% | -6.303e-04 | 4.284e-03 | 1.917e-02 | |  |  |
| 90%-10% | -2.127e-03 | -8.384e-03 | 1.734e-02 | |  |  |
| **label percentage 5%** | | | |  |  |  |
| 10%-90% | | -1.093e-01 | -1.580e-01 | -9.290e-02 | |  |
| 30%-70% | | -1.630e-03 | -3.806e-02 | -1.907e-02 | |  |
| 70%-30% | | -2.156e-04 | 1.801e-02 | 2.348e-02 | |  |
| 90%-10% | | -2.061e-02 | 4.526e-03 | 1.842e-02 | |  |
| **label percentage 1%** | | | |  |  |  |
| 10%-90% | -3.224e-02 | -1.314e-01 | -2.377e-01 | |  |  |
| 30%-70% | -2.609e-02 | -6.196e-02 | -3.227e-02 | |  |  |
| 70%-30% | 2.431e-02 | 6.662e-02 | 3.342e-02 | |  |  |
| 90%-10% | 5.016e-02 | 1.372e-01 | -6.119e-03 | |  |  |

MAE, Masked Autoencoder; PR-AUC, Precision Recall Area Under the Curve; SL, Supervised learning; VICReg, Variance-Invariance-Covariance Regularization.
